# Organic small-molecule NIR-II fluorophores for tumor phototheranostics

**DOI:** 10.1038/s41377-026-02212-w

**Published:** 2026-03-16

**Authors:** Dan Xiang, Zhichao Wang, Hongwei Zheng, Yuqi Tang, Quan Li

**Affiliations:** 1 Institute of Advanced Materials, School of Chemistry and Chemical Engineering, and School of Electronic Science & Engineering, Southeast University, Nanjing, 211189 China; 2 School of Intelligent Science and Engineering, Southeast University, Wuxi, 214026 China; 3 Materials Science Graduate Program, Kent State University, Kent, OH 44242 USA

**Keywords:** Optics and photonics, Applied optics

## Abstract

Near-infrared II (NIR-II) fluorophores possess transformative potential for biomedical applications, owing to their deep-tissue penetration, reduced tissue autofluorescence, and low phototoxicity. Recent breakthroughs in molecular engineering have accelerated the development of NIR-II organic small-molecule fluorophores based on versatile scaffolds, including cyanine, boron dipyrromethene, benzobisthiadiazole, xanthene, cyano-based derivatives, and small-molecule metal complexes. This review systematically summarizes the molecular engineering strategies, photophysical properties, and structure-function relationships of NIR-II fluorophores in the last five years. We highlight recent breakthroughs in their theranostic applications, including high-resolution deep-tissue imaging and efficient phototherapeutic modalities such as photodynamic and photothermal therapy. Finally, we present forward-looking perspectives on current challenges and emerging opportunities, aiming to provide insights for promoting continued innovation and clinical translation in this rapidly advancing field.

## Abstract (web summary)

This review systematically summarizes the recent advances in organic small-molecule NIR-II fluorophores for phototheranostics, providing insights into further innovation and facilitating clinical translation.

## Graphical abstract

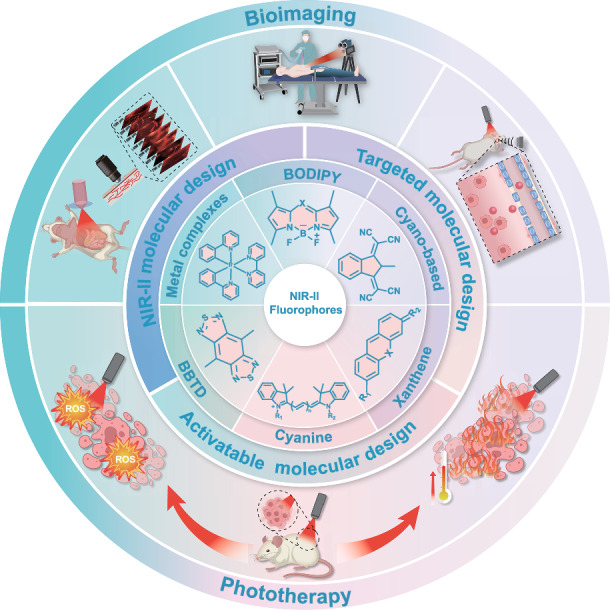

## Introduction

Cancer, one of the leading causes of mortality worldwide, poses a formidable threat to human health due to its complexity, diversity, and heterogeneity, which severely constrain clinical therapeutic efficacy^1–3^. In recent years, rapid advances in imaging agents and instrumentation have greatly promoted the application of optical and photonic technologies in modern medicine, particularly in the field of phototheranostics^4,5^. The core mechanism of phototheranostics lies in the utilization of phototherapeutic agents (PTAs) to absorb light energy and convert it into diverse forms of signals and energy. This conversion facilitates both disease diagnosis and therapy through photoacoustic (PA), photothermal, or photochemical effects^6^. This energy transduction enables multiple theranostic modalities. Diagnostic imaging techniques allow high-resolution visualization of biological processes and subcellular structures, forming the basis for precision diagnosis^7,8^. Therapeutic modalities, including photothermal therapy (PTT) and photodynamic therapy (PDT), induce targeted lesion damage. PTT leverages localized hyperthermia generated by photothermal agents, while PDT utilizes cytotoxic reactive oxygen species (ROS) generated by excited photosensitizers^9–11^. Synergistic strategies that integrate PDT and PTT can accelerate cell death, thereby enhancing therapeutic efficacy^12,13^. Compared to conventional therapies, phototheranostics exhibits pronounced advantages, including spatiotemporally controllable photodamage, non-invasiveness, high sensitivity, and minimal side effects, establishing it as a highly promising interventional approach for oncology^14^. Its applications have been widely extended from fundamental research to preclinical practice, encompassing biomarker detection, drug delivery monitoring, oncotherapy, and image-guided surgery^15–19^.

Despite the considerable promise of phototheranostics in biomedicine, its advancement remains confronted with significant challenges. The major limitation arises from the inherent optical opacity of biological tissues, which severely constrains the penetration depth of light^7^. Tissue heterogeneity induces substantial light scattering, while endogenous chromophores such as cytochromes, flavins, and melanin contribute to strong absorption and intrinsic autofluorescence, leading to elevated background noise and reduced imaging fidelity^20^. Crucially, the depth of light penetration exhibits a pronounced wavelength dependence, as light-tissue interactions (absorption and scattering) decrease with increasing wavelength^21^. Therefore, developing precise and efficient tumor theranostic technologies is of critical importance, as it can substantially improve patient survival and quality of life, reduce healthcare costs and societal burdens, and enhance global competitiveness in biomedicine^22–24^. However, conventional in vivo imaging modalities suffer from intrinsic drawbacks, including limited tissue penetration, low spatial resolution, and insufficient sensitivity, particularly when targeting deep tumors. Overcoming these limitations constitutes a pressing need for breakthrough solutions^2,25^.

Fluorescence imaging technology has emerged as a powerful tool in biomedical research, due to its high sensitivity, rapid response, and the absence of ionizing radiation^26–33^. Among the diverse optical imaging modalities, fluorescence imaging within the near-infrared II (NIR-II, 1000–1700 nm) window represents a major leap forward compared with those operating in the visible (400–700 nm) and near-infrared I (NIR-I, 700–1000 nm) regions^34–37^. Crucially, upon interaction with biological tissues, NIR-II light experiences significantly reduced absorption and scattering^38^. This fundamental property suppresses background noise, minimizes tissue autofluorescence, and results in negligible phototoxicity^39,40^. These intrinsic optical advantages enable NIR-II fluorescence imaging to achieve superior performance, including higher spatial resolution, greater penetration depth, enhanced signal-to-noise ratios (SNRs), and real-time capability, collectively offering a platform for the precise visualization and dynamic monitoring of deep-seated tumors^41^. Notably, the NIR-II window can be further divided into the NIR-IIa (1300–1400 nm), NIR-IIb (1500–1700 nm) sub-windows, with the latter typically offering even more advantageous imaging performance^39^.

The foremost challenge in achieving high-performance deep-tissue NIR-II imaging lies in the development of fluorescent probes that exhibit bright emission within this window^42,43^. Since the pioneering report of high-contrast NIR-II imaging in 2009, the field has witnessed remarkable progress, leading to the emergence of diverse probe materials, including carbon nanotubes^44,45^, quantum dots^46–48^, rare-earth-doped nanoparticles^49–51^, conjugated polymers^52^, and organic small-molecule dyes^53–58^. However, for successful clinical translation, NIR-II probes must satisfy rigorous requirements, such as high biocompatibility, low toxicity, and favorable in vivo clearance profiles^59,60^. Within this context, the long-term biosafety concerns associated with inorganic nanomaterials remain a major obstacle. In contrast, organic small-molecule fluorophores exhibit superior biocompatibility, precisely tunable molecular structures and photophysical properties (e.g., absorption/emission wavelengths, quantum yield, and metabolic pathways), as well as excellent tissue penetration, collectively offering the most promising prospects for clinical translation^61,62^.

Despite the significant advantages of organic small-molecule fluorescent probes, clinically approved agents available for tumor detection and therapy remain extremely limited (e.g., methylene blue, indocyanine green (ICG))^63–66^. However, their emission is restricted to the NIR-I region, imposing intrinsic limitations, including shallow tissue penetration and unsatisfactory stability^67^. Consequently, the development of novel organic small-molecule fluorophores exhibiting bright NIR-II emission, excellent biocompatibility, and strong potential for clinical translation has become an urgent priority^68–70^. Most small-molecule organic fluorophores are constructed around key structural scaffolds such as cyanine, boron dipyrromethene (BODIPY), benzobisthiadiazole (BBTD), xanthene, cyano-based derivatives and small-molecule metal complexes, which serve as their fundamental emissive cores^71,72^. Indeed, the majority of reported NIR-II organic small-molecule fluorophores incorporate one or more of these core scaffolds^73,74^. Their readily tunable photophysical properties and substantial potential for functionalization make them an ideal foundation for designing advanced NIR-II probes^75,76^. A comprehensive understanding of the strengths and limitations of these core fluorophores is therefore critical to their rational design and successful implementation in practical NIR-II imaging applications.

**Fig. 1:**
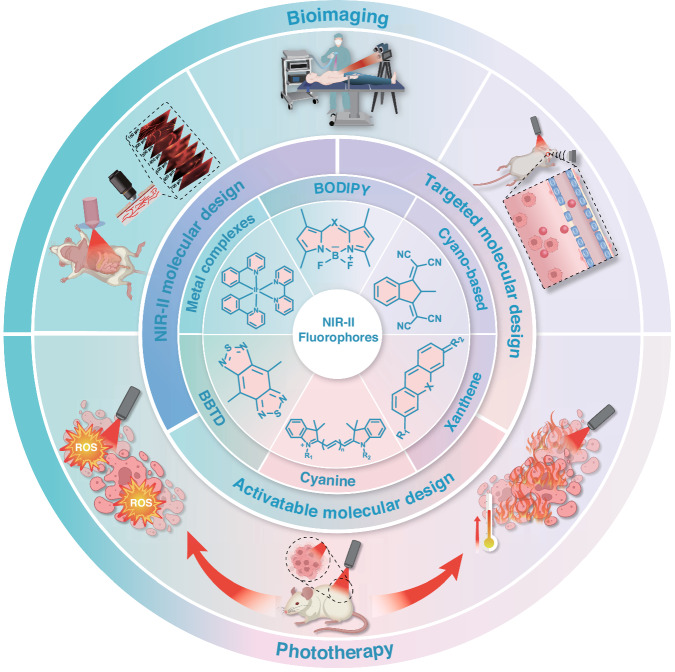
Overview of organic small-molecule NIR-II fluorophores for tumor phototheranostics

This review provides a systematic overview of recent advances in organic small-molecule NIR-II fluorophores, encompassing molecular design strategies, methodologies for optimizing photophysical properties, and applications in tumor theranostics (Fig. 1). By reviewing major progress and persisting challenges, we focus on breakthroughs in optimizing optical performance and in vivo phototheranostics applications. Ultimately, this review aims to provide strategic guidance and inspiration for researchers designing next-generation, high-performance NIR-II organic small-molecule fluorophores to accelerate their clinical translation.

## Molecular design

### Cyanine-based fluorophores

**Fig. 2:**
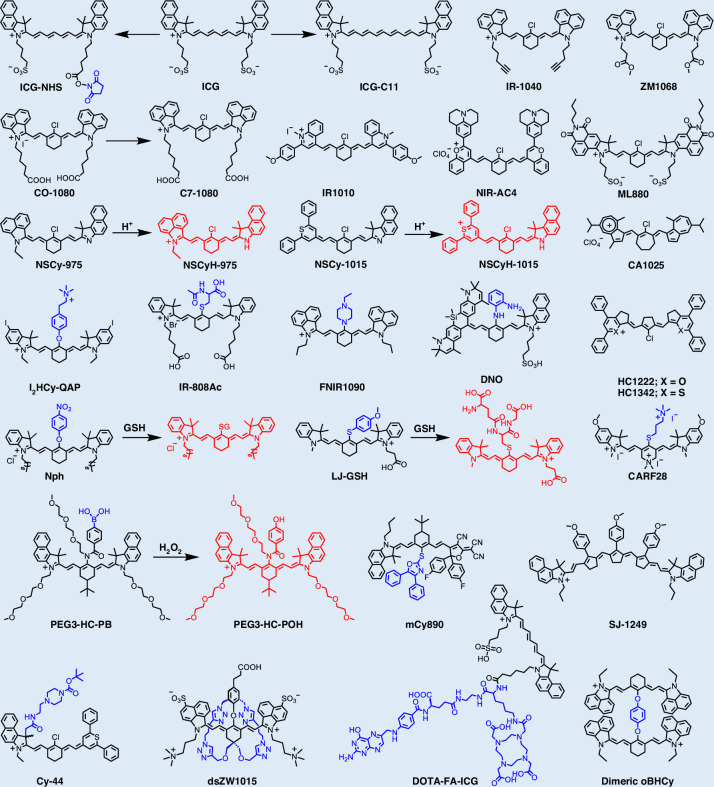
Chemical structures of organic small-molecule NIR-II cyanine-based structures

Cyanine dyes, particularly heptamethine cyanines and their derivatives (e.g., Cy7 and HCy dyes), represent one of the most prominent classes of near-infrared (NIR) fluorophores^77,78^. Owing to their high molar extinction coefficients, favorable quantum yields, excellent biocompatibility, and low systemic toxicity, these dyes are highly prominent in bioimaging and disease diagnosis (Fig. 2 and Table 1)^79–108^. For instance, ICG, the only Food and Drug Administration (FDA)-approved Cy7 dye, emits primarily within the NIR-I window (~ 800 nm)^109^. Although its emission tail extends beyond 1000 nm, suggesting potential for NIR-II imaging, its tissue penetration depth and SNR are inferior to those of dedicated NIR-II fluorophores^110^. This limitation underscores the urgent need to develop new cyanine derivatives with significantly red-shifted optical properties to fully exploit NIR-II imaging for deep-tissue diagnostics.

**Table 1:** NIR-II cyanine-based fluorophores

Fluorophore	*λ*_abs_ (nm)	*λ*_em_ (nm)	Quantum yield (%)	*ε* (M^−1^ cm^−1^)	Reference
IR-1040	1040	1069	0.12^a^ (DCM)	2.94 × 10^5^	^95^
ICG-C11	975	1078	2.1^a^ (DMSO)	5.48 × 10^4^	^92^
ZM1068	725	1050	0.08^a^ (H_2_O)	/	^81^
CO-1080	1044	1080	/	1.08 × 10^5^	^99^
C7-1080	1046	1089	/	/	^107^
IR-1010	1010	1058	3.08 (DCM)	2.33 × 10^5^	^94^
ML880	880	912	11.1^b^ (DMSO)	8.84 × 10^4^	^83^
NSCyH-975	975	1185	0.032^c^ (CPBS/MeCN)	/	^88^
NSCyH-1015	1015	1185	0.026^c^ (CPBS/MeCN)	/	^89^
CA1025	922	1025	0.05^a^ (CHCl_3_)	1.60 × 10^5^	^100^
I_2_HCy-QAP	795	820	/	/	^97^
IR-808Ac	787	/	2.57^a^ (PBS)	2.08 × 10^5^	^91^
NIR-AC4	1149	1206	0.063^a^ (DCM)	1.13 × 10^5^	^104^
FNIR-1090	817	1090	0.074^c^ (DMSO)	3.66 × 10^4^	^86^
DNO	742	1012	/	1.30 × 10^4^	^108^
HC1222	1180	1222	0.016^a^ (DCE)	1.17 × 10^5^	^85^
HC1342	1286	1342	0.015^a^ (DCE)	1.08 × 10^5^	^85^
Nph	786	808	/	/	^80^
LJ-GSH	780	815	/	/	^106^
CARF28	777	808	2.32 ^d^ (PBS)	1.14 × 10^5^	^103^
PEG3-HC-PB	830	950	/	/	^56^
mCy890	890	953	0.1^a^ (H_2_O)	1.00 × 10^5^	^93^
SJ-1249	1194	1249	0.048^a^ (MeOH)	2.44 × 10^5^	^101^
Cy-44	730	1020	0.05 ^d^ (H_2_O/MeCN/ATP)	/	^202^
dsZW1015	1030	1056	0.1^a^ (DMSO)	1.62 × 10^5^	^87^
DOTA-FA-ICG	790	820	/	/	^84^
dimeric oBHCy	878	1057	0.07^c^ (DMSO)	2.99 × 10^5^	^96^

^a^Quantum yield calculated with IR-26 as reference. ^b^Quantum yield calculated with ECXb as reference; ^c^Quantum yield calculated with IR-1061 as reference; ^d^Quantum yield calculated with ICG as reference. *DCM* Dichloromethane, *DMSO* dimethyl sulfoxide, *CPBS* citrate phosphate buffer solution, *CHCl*_*3*_ trichloromethane, *PBS* phosphate buffer solution, *MeOH* methanol, *MeCN* acetonitrile

#### Design strategies for NIR-II cyanine-based fluorophores

**Fig. 3:**
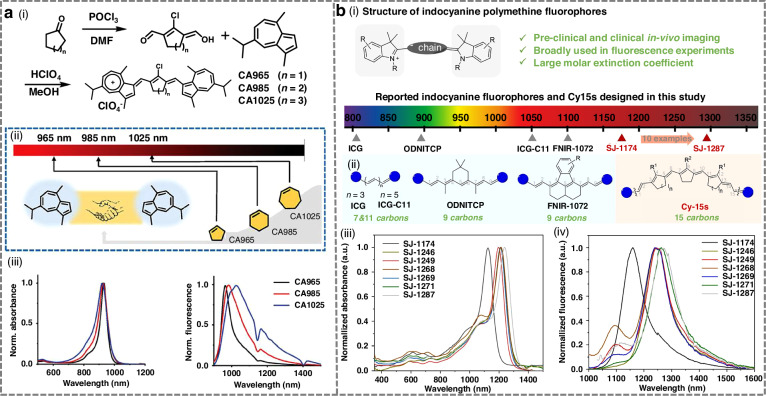
Design strategies for NIR-II cyanine-based fluorophores. **a** i) Synthetic route to cyazulenes (CA965, CA985, and CA1025). ii) Adjustment of cycloalkenes in NIR-II cyazulene fluorophores. iii) Normalization of absorption and emission spectra of CA965, CA985, and CA1025 in CHCl_3_. Reproduced with permission^100^. Copyright 2025, Wiley-VCH GmbH. **b** i) Structure of indocyanine polymethine fluorophores with indocyanine terminal groups. ii) Structures and emission wavelength of reported indocyanine polymethines and Cy15s reported here. The blue balls indicate indocyanine terminal groups, and the green numbers represent the number of carbon atoms in the conjugation chain. iii) Normalized absorption spectra of representative Cy15s (5 × 10^−6^ mol/L) in DCM. iv) Normalized emission spectra of representative Cy15s (5 × 10^−6^ mol/L) in deuterated DCM under 980 nm laser excitation. Reproduced with permission^101^. Copyright 2025, Elsevier Ltd

Significant red-shifts in emission wavelength are primarily achieved through two strategies: elongation of the polymethine chain and fine structural modifications, including extension or modification of terminal heterocycles, electron-donating or withdrawing substituents on terminal units and cyclization of the polymethine chain^111–113^. Shi et al. reported a novel series of azulene-based NIR-II fluorophores (Fig. 3a), with tunable cycloalkene units and spectroscopic properties^100^. These azulene derivatives were efficiently synthesized via the facile coupling of cycloalkenes with azulene derivatives. All compounds exhibited strong absorption within the NIR-II window and strong fluorescence emission (945–1025 nm). Zhang et al.^104^ engineered a series of novel dyes (NIR-ACs) via simple donor ectopic substitution at the terminal structures of NIR-II cyanine. Compared to the original NIR-II cyanine Flav7, these NIR-ACs exhibited markedly red-shifted emission (red-shifts of 87–263 nm), substantially larger Stokes shifts ( > 42 nm, up to 112 nm), and favorable fluorescence brightness. Their maximum emission extended beyond 1300 nm, with the spectral tail reaching above 1500 nm.

Zhang et al. developed indocyanine pentadecamethine fluorophores (Cy15s) via a π-chain elongation strategy, achieving ultra-long emission wavelengths and enhanced brightness (Fig. 3b)^101^. This design elegantly circumvented the inherent instability of reactive, π-extended, bifunctionalized polyene intermediates common in traditional syntheses. The resulting Cy15 fluorophores displayed profoundly red-shifted optical properties, with absorption and emission maxima at 1241 nm and 1287 nm, respectively. This emission represented the longest wavelength reported for an indocyanine polymethine dye.

#### Design strategies for activatable NIR-II cyanine-based fluorophores

The diagnostic precision of most NIR-II cyanine dyes is severely limited by their “always-on” fluorescence signature. This persistent emission, which is unresponsive to the microenvironment, results in high background noise, low imaging contrast, and poor specificity^88^. Compounding this issue, significant liver accumulation of these dyes generates intense background fluorescence, further compromising imaging accuracy^66^. This inevitably compromises the accuracy of disease diagnosis and therapy. Consequently, developing NIR-II cyanine dyes with large Stokes shifts, high stability, and effective suppression of hepatic background fluorescence is paramount for precise in vivo diagnostics.

**Fig. 4:**
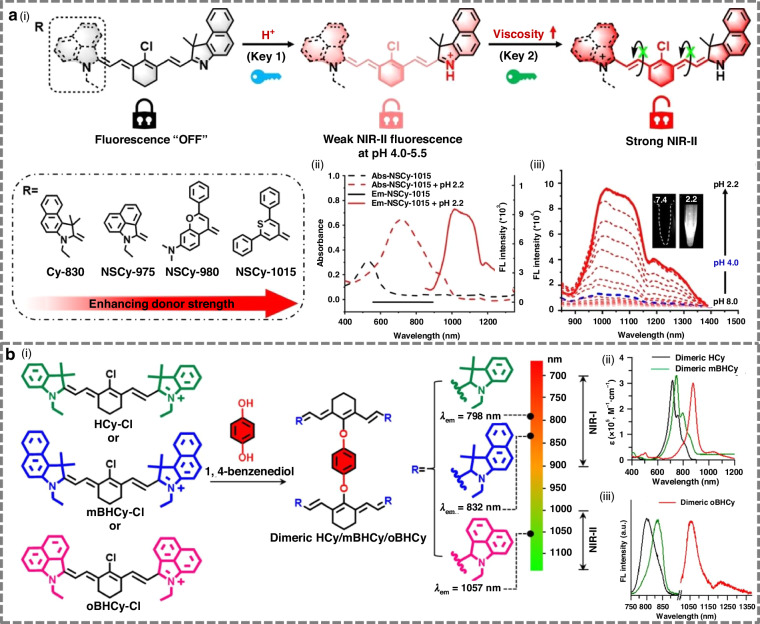
Design strategies for targeted cyanine-based fluorophores. **a** i) Schematic illustration of design strategy and response mechanism of NIR-II NSCyanine dyes to pH and viscosity in diseases. ii) NSCy-1015 in pH 7.4 and pH 2.2 CPBS buffer solutions (containing 50% CH_3_CN for dispersed solution). iii) NSCy-1015 in different pH buffer solutions (containing 50% CH_3_CN), λex = 808 nm. Insert: NIR-II fluorescence images of fluorophores in pH 7.4 and pH 2.2 buffer solutions (808 nm laser, 50 mW cm^−2^, 50 ms). Reproduced with permission^88^. Copyright 2023, Wiley-VCH GmbH. **b** i) The molecular design strategy of dimeric HCy photothermal transducers and molecular structures of dimeric HCy, dimeric mBHCy, and dimeric oBHCy. ii) Absorption spectra and iii) fluorescence emission spectra of dimeric HCy, dimeric mBHCy, and dimeric oBHCy all in DMSO (excited at 735 nm for NIR-I spectra and at 980 nm for NIR-II spectra). Reproduced with permission^96^. Copyright 2024, American Chemical Society

To address these limitations, Xiong et al. developed a pH/viscosity-activatable NIR-II non-symmetric cyanine (NSCyanine) dye, specifically engineered for high signal-to-background ratio (SBR) theranostics within the liver in vivo (Fig. 4a)^88^. Their fluorescence activation was based on a dual-environmental sensitivity mechanism: the dye remained in an “off” state at physiological pH, while protonation under acidic conditions (pH 4.0–5.5) triggered weak fluorescence, which was further amplified in high-viscosity environments, yielding up to a 20-fold signal enhancement. This dual-activation mechanism effectively minimized hepatic background and enhanced imaging precision.

Yu et al. reported a glutathione (GSH)-activatable NIR-II probe, LJ-GSH, which initially exhibited quenched fluorescence due to the weak electron-donating nature of its thiophenol group^106^. Within tumor cells and tissues, overexpressed GSH reacted specifically with LJ-GSH via an aromatic nucleophilic substitution reaction, leading to the formation of a thiol skeleton. The structural rearrangement significantly enhanced intramolecular charge transfer (ICT), thereby restoring strong fluorescence emission at 815 nm/910 nm. This GSH-responsive activation mechanism substantially improved the probe’s tumor-targeting specificity. Zhang et al. identified 2-mercapto-1,3,4-thiadiazole (MTD) as an H_2_S-responsive unit and conjugated it to a cyanine scaffold to create the ratiometric PA probe Cy-MTD^102^. Building upon this, they further fabricated a novel dual-ratiometric nanoprobe DCNP@Cy-MTD by integrating Cy-MTD onto a down-conversion nanoparticle (DCNP). This nanoprobe enabled the real-time dynamic quantification of H_2_S by simultaneously leveraging NIR-II fluorescence and NIR-I PA signals. Cy-MTD exhibited a strong absorption peak at 840 nm, with its absorption spectrum overlapping the 808 nm excitation band of the DCNP core. Consequently, when conjugated to the DCNP surface, Cy-MTD acted as an optical filter, quenching the DCNP’s 1550 nm emission (under 808 nm excitation) via competitive absorption quenching. In the presence of H_2_S, Cy-MTD underwent a specific molecular transformation, causing a significant blue-shift in its absorption peak from 840 nm to 670 nm. This spectral shift enabled the ratiometric PA imaging of H_2_S based on the signal ratio at 670 nm and 840 nm (PA_670_/PA_840_).

#### Design strategies for targeted cyanine-based fluorophores

Targeted molecular design represents a pivotal approach to overcoming the nonspecific accumulation and low selectivity that limit the in vivo performance of NIR-II cyanine fluorophores^103^. This strategy integrates biological recognition motifs or organelle-targeting elements into the dye architecture, enabling selective accumulation at desired sites, ranging from specific receptors and subcellular organelles to complex tumor microenvironments. Such targeted systems not only improve imaging precision and therapeutic efficiency but also minimize off-target effects. Feng et al. developed a ligand-based NIR-II imaging probe (hCG-ICG) by covalently conjugating indocyanine green N-hydroxysuccinimide ester to human chorionic gonadotropin (hCG), which specifically bound to luteinizing hormone receptors on ovarian follicles and tumor surfaces^105^. The combination of hCG-ICG for targeted follicle labeling and DCNPs for real-time circulatory visualization allowed dynamic dual-channel imaging of murine ovaries throughout developmental stages, estrous cycles, and induced ovulation.

Notably, several HCy dyes possess structure-inherent mitochondrial targetability due to their delocalized cationic cores and suitable lipophilicity, which enable efficient accumulation driven by the mitochondrial membrane potential. Li et al. reported a mitochondria-targeted dimeric cyanine dye (dimeric oBHCy), featuring two heptamethine cyanine moieties connected via a bisphenol linker at the central polymethine position (Fig. 4b)^96^. This distinctive molecular architecture endowed dimeric oBHCy with intense NIR-II fluorescence emission, a high photothermal conversion efficiency (PCE), and exceptional photostability. Dimeric oBHCy demonstrated the capability to precisely localize to cellular mitochondria and efficiently induce mitochondrial damage upon NIR irradiation.

To address the challenge of treating deep-seated pancreatic tumors, Ye et al. developed a NIR-II photoactivatable theranostic nanoplatform that targets carbonic anhydrase (CA), designated as IRNPs-SBA/Pt^IV^, for the synergistic combination of chemotherapy and NIR-II fluorescence imagingguided PTT^95^. By leveraging active targeting of CA, which is overexpressed in the tumor, IRNPs-SBA/Pt^IV^ achieved efficient accumulation in pancreatic tumor tissue.

### BBTD-based fluorophores

**Fig. 5:**
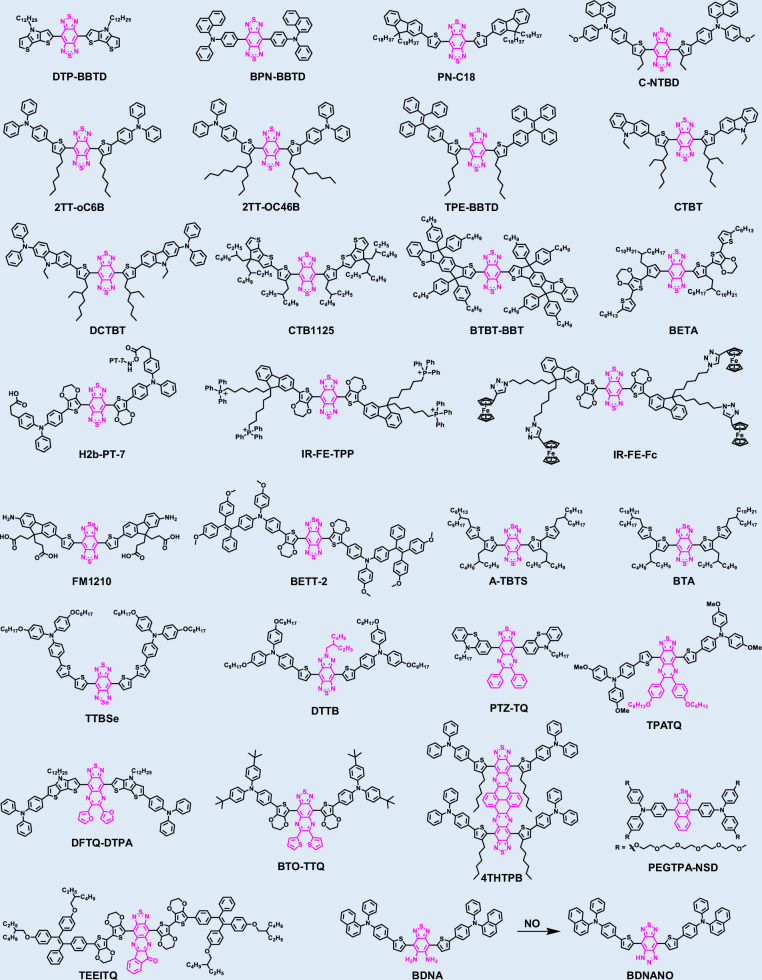
Chemical structures of organic small-molecule NIR-II benzobisthiadiazole-based fluorophores

The benzobisthiadiazole (BBTD) core and its derivatives are potent electron-accepting units with excellent quantum efficiency, making them ideal for the design of low-bandgap polymers and long-wavelength NIR-II fluorophores (Fig. 5 and Table 2)^114–150^. Their strong electron-withdrawing ability effectively promotes ICT when paired with appropriate electron donors. A common strategy for designing NIR-II small-molecule dyes involves embedding the chromophore within a donor-acceptor (D-A) framework^71^. In such designs, the synergistic interplay between a strong electron-withdrawing acceptor and a strong electron-donating donor significantly narrows the energy gap between the highest occupied molecular orbital (HOMO) and the lowest unoccupied molecular orbital (LUMO), thereby driving a red-shift in absorption wavelength into the NIR-II region. Further optimization has shown that symmetric donor-acceptor-donor (D-A-D) architectures are superior to D-A configurations for facilitating efficient ICT^151^. The conjugated linkage of the acceptor core to bilateral donors profoundly reduces the energy gap, thereby substantially enhancing its optical performance.

**Table 2:** NIR-II benzobisthiadiazole-based fluorophores

Fluorophore	*λ*_abs_ (nm)	*λ*_em_ (nm)	Quantum yield (%)	*ε* (M^−1^ cm^−1^)	Reference
DTP-BBTD	974	1230	/	/	^134^
BPN-BBTD	710	930	1.8^a^ (H_2_O)	/	^120^
PN-C18	849	1004	0.81 (CHCl_3_)	4.23 × 10^4^	^148^
C-NTBD	760	1100	0.22^a^ (DMSO)	1.69 × 10^4^	^128^
2TT-oC6B	733	1030	11^a^ (H_2_O)	/	^114^
TPE-BBTD	685	908	/	/	^136^
CTBT	669	863	/	1.75 × 10^4^	^130^
DCTBT	699	988	/	1.78 × 10^4^	^130^
CTB1125	895	1125	10.2^b^ (Hexane)	/	^141^
BTBT-BBT	947	1178	7.4^c^ (Toluene)	4.50 × 10^4^	^125^
BETA	873	1300	0.019 ^d^ (CHCl_3_)	1.13 × 10^4^	^129^
H2b-PT-7	808	1100	0.02^a^ (H_2_O)	/	^29^
IR-FE-Fc	808	1030	/	/	^133^
FM1210	980	1210	0.036^a^ (DCM)	/	^115^
BETT-2	915	1240	0.13^a^ (DCM)	1.81 × 10^3^	^139^
α-TBTS	940	1134	1.2^a^ (THF)	/	^145^
BTA	947	1108	/	/	^137^
TTBSe	1007	1250	/	/	^149^
DTTB	760	985	13.4^d^ (H_2_O/THF)	/	^116^
PTZ-TQ	660	1250	/	/	^123^
TPATQ	789	1078	/	/	^122^
DFTQ-DTPA	922	1127	0.064^a^ (Toluene)	/	^131^
4THTPB	793	1058	0.52^a^ (THF)	/	^142^
PEGTPA-NSD	590	833	/	/	^147^
TEEITQ	808	> 1000	/	/	^144^

^a^Quantum yield calculated with IR-26 as reference; ^b^Quantum yield calculated with ICG as reference; ^c^Quantum yield calculated with FT-BBT as reference; ^d^Quantum yield calculated with IR-1061 as reference. *THF* tetrahydrofuran

#### Design strategy for NIR-II BBTD-based fluorophores

**Fig. 6:**
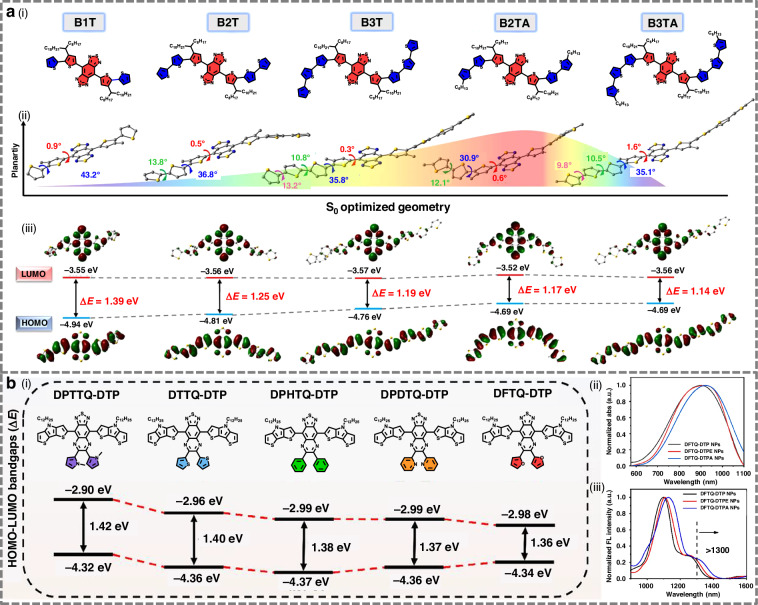
Design strategy for NIR-II benzobisthiadiazole-based fluorophores. **a** i) Chemical structures, ii) optimized ground-state (S_0_) geometries, and iii) the HOMO and LUMO distributions of B1T, B2T, B3T, B2TA, and B3TA. Reproduced with permission^129^. Copyright 2023, Wiley-VCH GmbH. **b** i) HOMO-LUMO (highest occupied molecular orbitals-lowest unoccupied molecular orbitals) bandgaps of the fluorophores containing different thiadiazolo quinoxaline-based acceptors. ii) Normalized absorption spectra of the NPs in water. iii) Normalized emission spectra of the NPs in water. Reproduced with permission^131^. Copyright 2023, Wiley-VCH GmbH

Recent studies have demonstrated that higher molecular planarity, stronger donor strength, and increasing acceptor electron affinity in BBTD-based systems can synergistically enhance conjugation, ICT strength, and excited-state stability, which collectively result in narrowed bandgaps and superior NIR-II optical performance^117–121^. Fan et al. proposed a strategy based on donor and side-chain engineering to modulate molecular planarity, which enabled the successful design of high-performance NIR-II fluorophores (Fig. 6a)^129^. Using the highly planar and low-bandgap acceptor BBTD, they coupled it with planar thiophene donors to extend π-conjugation. Increasing the number of thiophene units induced pronounced red-shifts in absorption into the NIR-II region, while moderate side-chain substitution further enhanced fluorescence brightness and PCE by maintaining an optimal conformation.

To overcome tissue penetration limitations in deep-brain imaging, Wang et al. designed a series of D-A-D molecules based on BBTD cores^130^. Ortho-alkyl-substituted thiophene units were introduced on both sides of BBTD, serving dual functions as electron donors and π-spacers. By gradually increasing donor strength from carbazole to diphenylamine-substituted thieno[3,2-b]indole, they achieved stepwise narrowing of the HOMO-LUMO gap and marked red-shifts in absorption and emission. The introduction of twisted, rotatable diphenylamine units not only enhanced ICT but also imparted aggregation-induced emission (AIE) behavior, leading to high fluorescence quantum yields. Compared to aggregation-caused quenching (ACQ)-type dyes, which suffer from π–π stacking-induced non-radiative decay, AIE luminogens become more emissive upon aggregation because the restriction of intramolecular motion (RIM) suppresses these non-radiative pathways. This distinction explains why AIE-active NIR-II dyes exhibit superior brightness and stability in biological environments.

The electron affinity of 6,7-diphenyl-[1,2,5]thiadiazolo[3,4-g]quinoline and its analogs is slightly weaker than that of BBTD, but they possess excellent chemical stability and enhanced solubility, demonstrating significant potential for the development of NIR-II fluorophores^152^. Wu et al. designed a novel NIR-II fluorophore with a D-A-D architecture, employing the strong electron-donating unit dithieno[3,2-b:2’,3’-d]pyrrole (DTP) as the donor and 6,7-di(furan-2-yl)-[1,2,5]thiadiazolo[3,4-g]quinoxaline (DFTQ) as the acceptor (Fig. 6b)^131^. The hydrophobic DFTQ-DTP fluorophore was encapsulated with the amphiphilic polymer DSPE-PEG2000 to form water-dispersible nanoparticles. The resulting NPs exhibited exceptional NIR-II fluorescence, with an emission spectrum predominantly in the NIR-II region and a significant extension into the >1300 nm sub-window.

#### Design strategies for activatable NIR-II BBTD-based fluorophores

To enable precise detection of specific biomolecules, researchers have developed activatable BBTD-based probes whose fluorescence can be switched “on” in response to target-induced chemical reactions. Wu et al. reported an activatable molecular probe, featuring an ortho-phenylenediamino moiety as a specific NO-responsive unit attached to the benzothiadiazole (BTD) core^124^. Upon reaction with NO, the phenylenediamino group was converted into a triazole, markedly enhancing the electron-withdrawing strength of the BTD core and activating strong NIR-II fluorescence (900–1100 nm) and optoacoustic absorption (650–850 nm). Furthermore, Wu et al. developed a ratiometric near-infrared fluorescence (NIRF) and PA dual-modal nanoprobe RAPNP for NO detection^134^. A key design feature was the synergistic integration of two complementary fluorophores: a NO/acid dual-responsive molecule (DTP-BTDA) and a nonresponsive fluorophore (DTP-BBTD). Both fluorophores incorporated a dithienopyrrole (DTP) unit as a strong electron donor. The DTP unit conferred a pronounced ICT effect, which enabled long-wavelength NIR emission crucial for deep-tissue imaging. The core functionality of the probe hinged on the design of DTP-BTDA. In DTP-BTDA, the BTDA moiety can be rapidly oxidized by NO in a weakly acidic environment, enabling the activation of NIRF and PA signals.

### BODIPY-based fluorophores

**Fig. 7:**
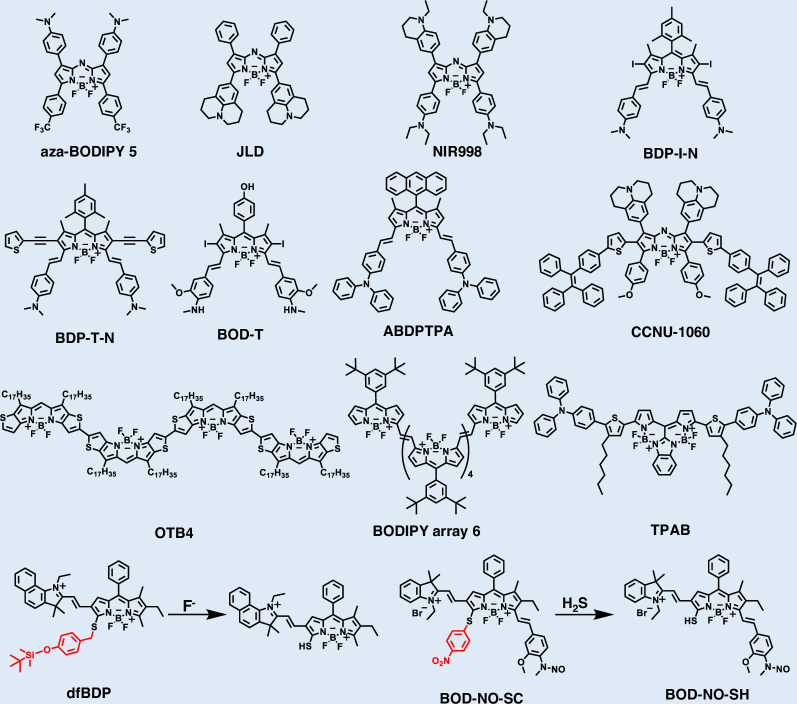
Chemical structures of organic small-molecule NIR-II BODIPY-based fluorophores

BODIPY derivatives are an emerging class of small-molecule fluorophores that are key components in NIR dyes due to their strongly electron-withdrawing BF_2_ group and exceptional stability (Fig. 7 and Table 3)^153–168^. The typical BODIPY core consists of two pyrrole rings linked by a methine bridge at the meso-carbon atom, forming an extended π-conjugated system^153^. The hydrogen at the meso-position can be substituted with various groups, while a central boron atom bridges the α-positions adjacent to the pyrrole nitrogens. Both BODIPY-based NIR dyes and their BODIPY analogs exhibit high molar absorptivity, facile derivatization, and excellent photostability^169^.

**Table 3:** NIR-II BODIPY-based fluorophores

Fluorophore	*λ*_abs_ (nm)	*λ*_em_ (nm)	Quantum yield (%)	*ε* (M^−1^ cm^−1^)	Reference
aza-BODIPY 5	850	1000	4.3^a^ (DMSO)	5.73 × 10^4^	^164^
JLD	880	968	0.3 (DCM)	1.85 × 10^5^	^162^
NIR998	859	998	/	/	^157^
BDP-I-N	747	1000	12.3^b^ (Toluene)	4.04 × 10^4^	^156^
BDP-T-N	772	1000	27.6^b^ (H_2_O)	1.09 × 10^5^	^155^
BOD-T	700	910	0.58^c^ (H_2_O)	/	^168^
ABDPTPA	708	1000	/	/	^159^
CCNU-1060	877	1060	0.25^c^ (CHCl_3_)	3.1 × 10^4^	^154^
OTB4	1169	1201	/	5.80 × 10^5^	^161^
BODIPY array 6	1114	1136	0.2^d^ (Toluene)	1.08 × 10^5^	^153^
TPAB	858	945	0.83^c^ (H_2_O)	1.01 × 10^5^	^166^
dfBDP	528	611	/	/	^163^
BOD-NO-SC	588	655	/	/	^158^
BOD-NO-SH	806	936	0.06 (PBS/MeCN)	/	^158^

^a^Quantum yield calculated with ICG as reference; ^b^Quantum yield calculated with single-walled carbon nanotubes as reference; ^c^Quantum yield calculated with IR-26 as reference; ^d^Quantum yield calculated with IR-1061 as reference

#### Design strategies for NIR-II BODIPY-based fluorophores

**Fig. 8:**
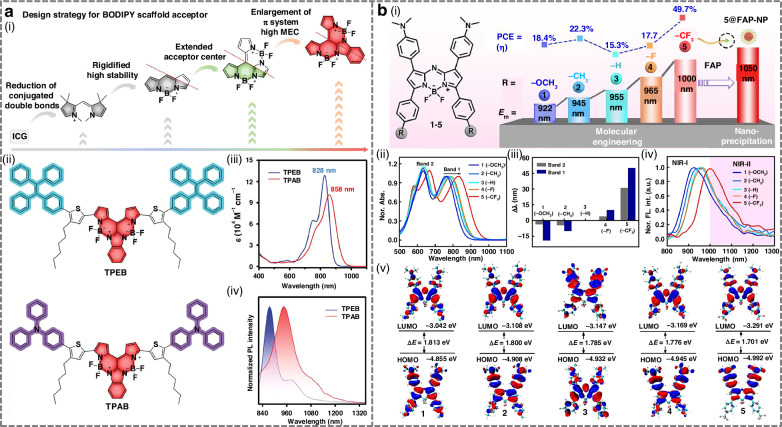
Design strategies for NIR-II BODIPY-based fluorophores. **a** i) Design strategy for BOIMPY acceptor. ii) The chemical structure of TPEB and TPAB. iii) The absorption spectra and iv) emission spectra in toluene of TPEB and TPAB (10 μM). Reproduced with permission^166^. Copyright 2025, Wiley-VCH GmbH. **b** i) Molecular engineering and photophysical properties of the BODIPY derivatives 1–5. ii) Normalized absorption spectra of compounds 1–5, 10 µM in dichloromethane. iii) Wavelength shifts of the BODIPY derivatives (1, 2, 4, 5) compared with compound 3 as a reference. iv) Normalized fluorescence spectra of compounds 1–5, 10 µM in dichloromethane, excited at 808 nm. v) Frontier molecular orbitals and relative energies of compounds 1–5. Reproduced with permission^164^. Copyright 2024, Wiley-VCH GmbH

Two main strategies extend BODIPY absorption and emission into infrared regions: extending π-conjugation through structural enlargement, and substituting core heteroatoms such as replacing meso-carbon with nitrogen in BODIPYs^169^. Zhang et al. designed a BODIPY-based agent by adopting a D-A-A’ motif, where strong electron-withdrawing groups were introduced at the 3,5-positions (Fig. 8b)^164^. This push-pull modification more effectively red-shifted absorption and emission than conventional D-A-D’ systems. The optimized compound 5 displayed a distorted conformation, with NIR-II fluorescence maxima at 1000 nm in organic solvents and further shifted to 1050 nm in aqueous nanoparticles.

Tang et al. employed AIE to construct BOIMPY-based luminogens (Fig. 8a)^166^. The bis-(borondifluoride)-8-imidazodipyrromethene (BOIMPY) unit acted as a rigid, strongly electron-withdrawing acceptor, while tetraphenylethylene and triphenylamine served as donors and intramolecular rotors. This design suppressed π–π aggregation and enabled efficient NIR-II emission. The resulting dyes, TPEB and TPAB, showed high molar extinction coefficients and strong quantum yields, with TPEB nanoparticles achieving NIR-II imaging-guided photothermal ablation. Hao et al. expanded the BODIPY π-conjugation length through a controllable Stille cross-coupling strategy, yielding ethene-bridged arrays from dimers to hexamers^153^. Progressive extension of conjugation systematically red-shifted absorption across the NIR-II window (702–1114 nm). These arrays combined superior light-harvesting efficiency, robust photostability, and efficient photothermal conversion, underscoring the power of π-conjugation expansion as a red-shift design principle.

#### Design strategies for activatable NIR-II BODIPY-based fluorophores

The design of responsive BODIPY-based fluorophores generally relies on structural modifications that confer selective reactivity toward specific stimuli while tuning their optical properties within the NIR window. Zhao et al. developed a dual-stimulus-responsive probe (BOD-NH-SC) that integrated an N-methyl-2-methoxyaniline moiety for selective NO recognition and a 4-nitrophenylethanol-substituted BODIPY group for H_2_S detection^158^. The probe exhibited reversible fluorescence switching between NIR-I (655 nm) and NIR-II (936 nm) emissions, enabling precise monitoring of the alternating presence of NO and H_2_S in living cells. This design highlighted the principle of installing orthogonal reactive motifs on the BODIPY scaffold to achieve ratiometric and reversible response behavior.

Qu et al. designed a photo-triggered platform (BOD-D) by integrating an aryl N-nitrosamine (NO donor) into an iodine-functionalized BODIPY scaffold^168^. Under 630 nm irradiation, BOD-D exhibited bright NIR-I fluorescence and efficient singlet oxygen (^1^O_2_) generation, whereas ultraviolet (UV) irradiation triggered NO release via N-N bond photolysis. Photolysis of the electron-withdrawing nitrosamine caused homolytic cleavage, releasing NO and generating an aniline radical. The radical underwent oxidation followed by in situ reduction, forming the aniline derivative BOD-T. The transformation from an electron-withdrawing nitrosamine to an electron-donating aniline derivative (BOD-T) induced strong ICT, leading to red-shifted absorption, efficient photothermal conversion, and bright NIR-II emission.

### Xanthene-based fluorophores

**Fig. 9:**
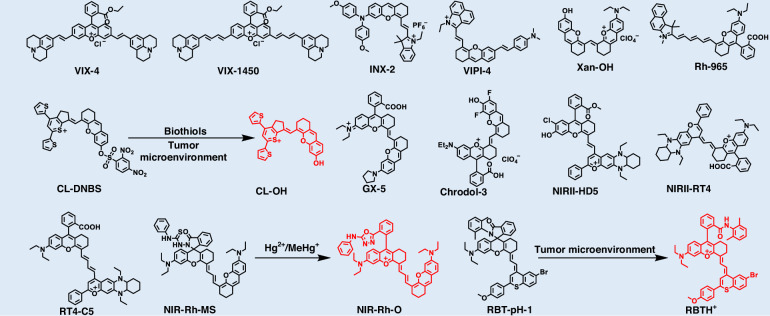
Chemical structures of organic small-molecule NIR-II xanthene-based fluorophores

Xanthene dyes, particularly rhodamines and fluoresceins, have attracted considerable attention in biomedical imaging owing to their outstanding photophysical properties, including high molar absorptivity, excellent photostability, facile functionalization, and tunable emission (Fig. 9 and Table 4)^170–184^. The xanthene scaffold consists of three fused rings: a central oxygen-containing six-membered heterocycle symmetrically flanked by benzene rings^170^. The primary reactivity is located at the C9 position, often functionalized with spirocycles or electron-donating groups, whereas the 3, 6-positions typically carry amino (-NH_2_) or hydroxy (-OH) substituents, yielding classical fluorophores such as rhodamines and fluoresceins. Strategic substitution at the C10 heteroatom (e.g., O, S, Si, P) has emerged as a powerful approach for tuning the optoelectronic properties of xanthene scaffolds^183^. Replacing oxygen with sulfur enhances resonance interactions, thereby reducing the energy gap and inducing red-shifted absorption and emission. Si or P substitution increases σ*-π* orbital participation and decreases the LUMO level, which also leads to red-shifted optical transitions. The rigid xanthene core imparts long emission wavelengths, excellent photostability, and high fluorescence quantum yields, providing an ideal platform for the development of NIR-II fluorescent fluorophores^175^.

**Table 4:** NIR-II xanthene-based fluorophores

Fluorophore	*λ*_abs_ (nm)	*λ*_em_ (nm)	Quantum yield (%)	*ε* (M^−1^ cm^−1^)	Reference
VIX-4	1028	1180	0.026^a^ (CHCl_3_)	2.34 × 10^5^	^170^
VIX-1450	1098	1450	0.023^a^ (DCM)	/	^175^
INX-2	735	820	/	/	^178^
VIPI-4	815	1056	0.02 (DMSO)	3.70 × 10^4^	^179^
Xan-OH	860	896	0.18^a^ (MeOH)	5.41 × 10^4^	^172^
Rh-965	915	965	0.4^b^ (EtOH)	8.24 × 10^4^	^170^
CL-DNBS	800	950	0.41^a^ (DMSO/PBS)	3.69 × 10^4^	^180^
CL-OH	920	1050	0.15^a^ (DMSO/PBS)	1.97 × 10^4^	^180^
GX-5	875	/	0.424^a^ (MeOH)	3.04 × 10^4^	^184^
Chrodol-3	870	902	0.34^a^ (MeOH)	4.00 × 10^4^	^172^
NIRII-HD5	854	936	0.28^a^ (PBS/EtOH)	1.03 × 10^5^	^173^
NIRII-RT4	856	989	1.42^a^ (DCM)	2.81 × 10^3^	^171^
RT4-C5	955	1000	/	/	^182^
NIR-Rh-O	970	1015	/	3.10 × 10^4^	^177^
RBT-pH-1	769	1020	/	/	^181^

^a^Quantum yield calculated with IR-26 as reference. *EtOH* ethyl alcohol

#### Design strategies for NIR-II xanthene-based fluorophores

The absorption and emission properties of xanthene-based fluorophores can be finely tuned by extending their π-conjugated systems and modulating D-A interactions^183^. A general rule, as exemplified in cyanine dyes, is that each methylene unit inserted between donor and acceptor typically induces a red-shift of ~100 nm^111–123^. Although each added methylene unit induces a ~100 nm red-shift, this strategy eventually reaches a plateau because excessively long polymethine chains become conformationally flexible and highly susceptible to non-radiative decay. These factors lead to decreased photostability, lower quantum yields, and broadened absorption bands, preventing further effective extension into the deep NIR-II region. To overcome these constraints, π-conjugation extension and donor-strength enhancement have been widely employed in xanthene scaffolds.

Leveraging the intrinsic D-A-D symmetry of xanthenes, Cui et al.^175^ designed VIX1250 and VIX1450, which exhibited emission peaks at 1250 and 1450 nm, respectively, both falling within the NIR-II window. Their red-shifted absorption (up to 1098 nm) enabled deep-tissue imaging with reduced autofluorescence and light-induced toxicity. Similarly, Ma et al. reported a series of xanthene dyes VIXs by introducing para-substituted styryl groups of progressively stronger electron-donating ability^170^. Among them, julolidine-substituted VIX-4 showed the most pronounced red-shift and significantly enhanced brightness, reaching emission at 1210 nm. Importantly, VIX-4 exhibited a large Stokes shift and six-fold higher brightness compared with classical BBTD dyes, enabling high-speed blood flow imaging in mice at up to 200 fps.

#### Design strategies for activatable NIR-II xanthene-based fluorophores

**Fig. 10:**
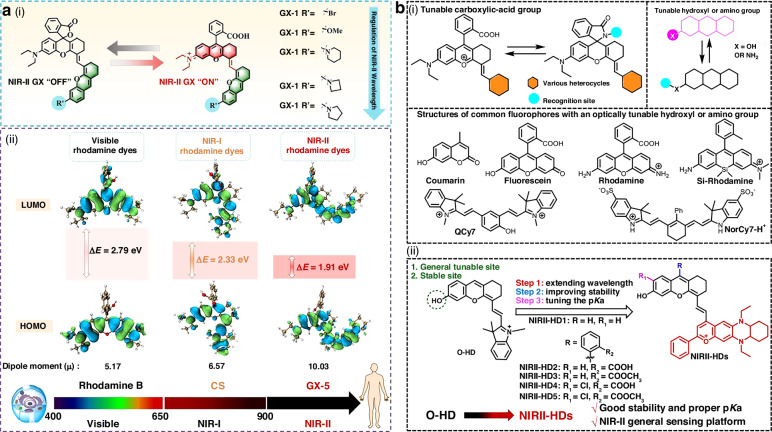
Design strategies for activatable NIR-II xanthene-based fluorophores. **a** i) Structures of the newly designed rhodamine-based NIR-II PA dyes GX. ii) The theoretical calculations of HOMO-LUMO gaps for rhodamine B, CS, and GX-5. Reproduced with permission^184^. Copyright 2024, Wiley-VCH GmbH. **b** i) The reported activated probes platform. ii) Rational design of the novel activated probes platform NIRII-HD dyes. Reproduced with permission^173^. Copyright 2022, Wiley-VCH GmbH

Recent progress has demonstrated that rational molecular engineering of the xanthene scaffold can effectively endow dyes with responsive and activatable properties^173,184^. One representative strategy is integrating a tunable carboxylic-acid group into the xanthene core. Lin et al. established the GX dye platform by coupling the carboxy-controlled spirocyclization of rhodamine dyes with NIR-II absorption/emission characteristics (Fig. 10a)^184^. Substituent engineering on the xanthene framework enabled systematic spectral red-shifting, and the optimized GX-5 scaffold exhibited absorption/emission maxima at 1082/1360 nm. Importantly, its modularity allowed the construction of a carbon monoxide-activatable probe (GX-5-CO), which achieved high-contrast NIR-II PA/FL imaging of endogenous CO in a murine hypertension model. A complementary approach leverages controllable hydroxyl or amino groups to construct activatable probes.

Yuan et al. developed hydroxyl-tuned NIR-II dyes (NIRII-HDs) by replacing the indole heterocycle of O-HD with an electron-rich decahydroquinoline benzopyran, introducing a protective benzoate group, and modulating the pKa of the phenolic hydroxyl group (Fig. 10b)^173^. This scaffold reconfiguration significantly improved dye stability and emission performance, with NIRII-HD displaying a 230 nm red-shift compared with O-HD and extending emission beyond 1200 nm. Building on the optimized NIRII-HD5, the team further constructed target-activatable probes for GSH, peroxynitrite, and alkaline phosphatase, enabling selective and reliable longitudinal biomarker monitoring in disease models.

### Cyano-based fluorophores

**Fig. 11:**
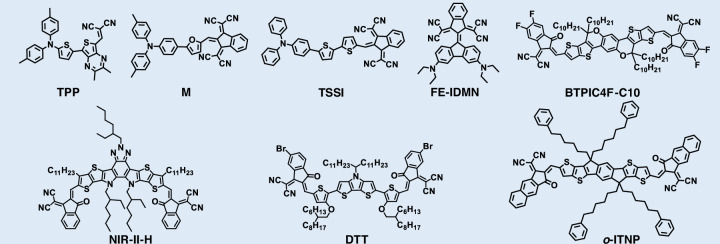
Chemical structures of organic small-molecule NIR-II cyano-based fluorophores

Cyano derivatives, such as malononitrile, are essential electron acceptors for designing NIR-II fluorescent probes (Fig. 11 and Table 5)^185–192^. The strong electron-withdrawing capability of cyano groups enables effective modulation of dye energy levels, leading to red-shifted absorption/emission and improved fluorescence quantum yields^186^. These configurations significantly narrow the molecular bandgap, shifting absorption and emission from the visible or NIR-I to the NIR-II region and thus fulfilling the requirements for deep-tissue imaging^185^. Strong D-A interactions enhance ICT, making the optical properties highly tunable^188^. These structural features also impart AIE characteristics, endowing the resulting AIE nanoparticles with high generality and extended emission wavelengths^191^.

**Table 5:** NIR-II cyano-based fluorophores

Fluorophore	*λ*_abs_ (nm)	*λ*_em_ (nm)	Quantum yield (%)	*ε* (M^−1^ cm^−1^)	Reference
TTP	683	802	0.21^a^ (DCM)	/	^188^
M	710	1000	/	/	^191^
TSSI	650	1000	/	/	^185^
FE-IDMN	855	1080	0.066^b^ (DCE)	1.17 × 10^4^	^187^
BTPIC4F-C10	640	967	7.9 (H_2_O)	/	^190^
NIR-II-H	750	800	0.66^c^ (H_2_O)	/	^186^
DTT	844	944	2.09^b^ (Toluene)	1.25 × 10^5^	^192^
o-ITNP	760	942	5.6^c^ (H_2_O)	/	^189^

^a^Quantum yield calculated with IR-1061 as reference; ^b^Quantum yield calculated with IR-26 as reference; ^c^Quantum yield calculated with ICG as reference. *DCE* ichloroethane

#### Design strategies for NIR-II cyano-based fluorophores

**Fig. 12:**
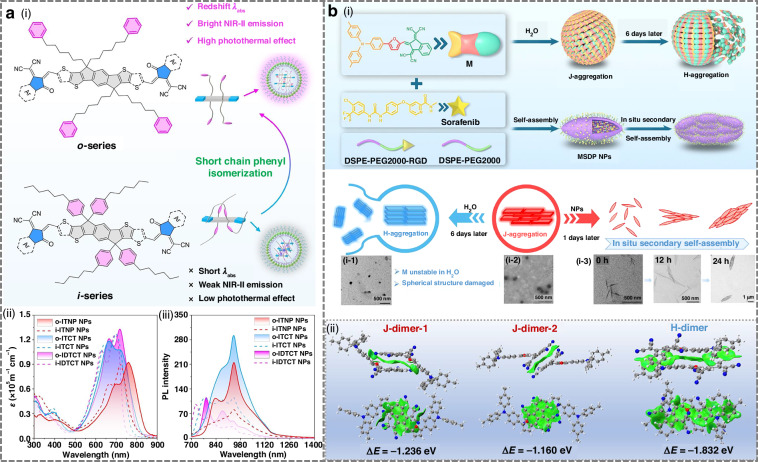
Design strategies for NIR-II cyano-based fluorophores. **a** i) Schematic diagram of side-chain phenyl isomerization-induced spatial conjugation and backbone interlocking for simultaneous enhancement of photothermal properties and NIR-II emission. ii) Ԑ and iii) photoluminescence (PL) spectra of six fluorophore NPs (Ex = 660 nm laser). Reproduced with permission^189^. Copyright 2025, Wiley-VCH GmbH. **b** i) the preparation and in situ self-assembly process of MSDP NPs. ii) The independent gradient model based on the Hirshfeld partition analysis of the dimer of M. Reproduced with permission^191^. Copyright 2025, Wiley-VCH GmbH

Tang et al. demonstrated that introducing thiophene units into conjugated backbones such as TSI and TSSI enhanced electron-donating ability, leading to significant red-shifts in absorption and emission^185^. Wang et al. devised a phenyl side-chain isomerization approach in six A-D-A fluorophores (o-ITNP, i-ITNP, o-ITCT, i-ITCT, o-IDTCT, i-IDTCT) (Fig. 12a)^189^. While o- and i-isomers shared similar absorption/emission profiles, o-series fluorophores exhibited brighter NIR fluorescence and improved photothermal conversion. This enhancement was attributed to the conformational restriction introduced by the ortho-phenyl substitution, which facilitated conformational locking and spatial conjugation between the side chain and the backbone. The consequent tighter molecular packing and extended π-conjugation plane enhanced electronic coupling and reduced the HOMO-LUMO gap. These structural changes accelerated both radiative and non-radiative decay pathways, resulting in red-shifted absorption, superior brightness, and enhanced photothermal performance. Nanoparticles derived from o-series consistently displayed red-shifted absorption, enhanced NIR-II brightness, and superior imaging-guided tumor ablation. Compact cyano-based D-A molecules can leverage through-space charge transfer (TSCT) to achieve long-wavelength emission upon aggregation. TSCT occurs when donor and acceptor units are positioned in close spatial proximity but are not directly connected through a conventional π-conjugated pathway. Instead, charge transfer takes place via orbital overlap in space, enabling efficient intramolecular electron redistribution. This spatially mediated charge transfer lowers the excited-state energy, narrows the optical bandgap, and leads to red-shifted NIR-II absorption/emission. For instance, short-conjugated D-A molecule TTP showed visible absorption and NIR-I emission as a monomer, but upon nanoparticle formation, efficient TSCT induced NIR-II emission extending to 1400 nm^188^. Such aggregation-induced red-shifts can also arise from the formation of J-aggregates, in which molecules adopt a slip-stacked arrangement that produces a lower-energy excitonic state through coherent exciton coupling. This configuration narrows the excitonic bandgap, leading to a characteristic bathochromic shift in absorption and emission together with enhanced oscillator strength. Li et al. developed a cyano-based donor-π-acceptor photosensitizer (M), featuring triphenylamine (donor) and 1,3-bis(dicyanomethylene)indane (acceptor) (Fig. 12b)^191^. Its self-assembly into J-aggregates led to a ~ 30 nm red-shift in absorption (from 681 nm to 710 nm), yielding strong NIR-II emission that reached 1350 nm.

### Small-molecule metal complexes

**Fig. 13:**
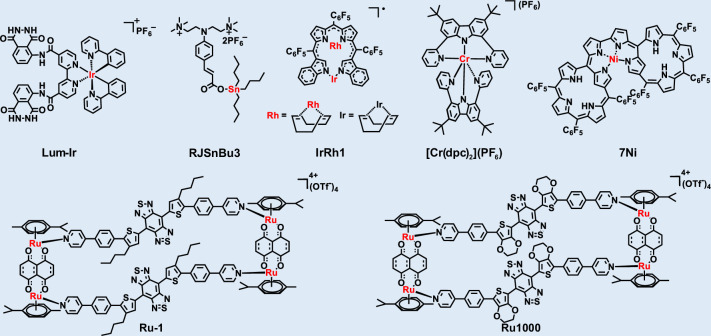
Chemical structures of organic small-molecule metal complex

Metal complexes are promising photofunctional materials due to their superior photostability, large Stokes shifts, and high emission quantum yields compared to organic chromophores (Fig. 13 and Table 6)^193–199^. Nevertheless, their biomedical application is hindered by a reliance on high-energy, short-wavelength excitation, which leads to phototoxicity and poor tissue penetration. Consequently, research efforts are now focused on developing metal complexes that emit in the NIR region, especially within the NIR-II window, which is ideal for deep-tissue imaging. Current design strategies to achieve redshifts can be categorized into five main approaches: (i) structural modulation of tetrapyrrolic complexes; (ii) coordination of metals to predesigned NIR-absorbing ligands; (iii) fine-tuning of metal-metal interactions; (iv) construction of coordination polymers; and (v) molecular engineering of bis(dithiolene) complexes^200^.

**Table 6:** NIR-II small-molecule metal complexes

Fluorophore	*λ*_abs_ (nm)	*λ*_em_ (nm)	Quantum yield (%)	*ε* (M^−1^ cm^−1^)	Reference
RJSnBu3	325-375	575	/	/	^196^
IrRh1	1415	/	/	/	^199^
[Cr(dpc)_2_](PF_6_)	425	1067	/	/	^194^
7Ni	1100	/	/	/	^193^
Ru-1	763	990	0.24^a^ (DCM)	3.87 × 10^4^	^197^
Ru1000	600-900	1000	0.94^a^ (DMSO)	/	^195^
Lum-Ir	385	615	/	/	^198^

^a^QY calculated with IR-26 as reference

Traditional Cr(III) polypyridyl complexes typically emit in the 727–778 nm range, limited to the red/NIR-I region. To overcome this, Wenger et al. developed a strategy centered on increasing metal-ligand bond covalence rather than merely optimizing the ligand field strength^194^. By employing tridentate ligands with a central π-donor amine and σ-donor/π-acceptor pyridines, they created the homoleptic Cr(III) complex Cr(dpc)_2_. This complex exhibited a dominant emission peak at 1067 nm, extending into the NIR-II region, a significant red-shift from conventional Cr(III) polypyridyl complexes. Furuta et al.^193^ demonstrated that metal-directed assembly of porphyrinoid dimers can also achieve NIR-II absorption. Their design employed a precisely modified N-confused porphyrin (NCP) unit providing peripheral metal-coordination sites. In the presence of Ni(II) salts, a metal-directed oxidative C-C coupling reaction linked the meso-pyrrolic α-positions, producing Ni(II)-coordinated NCP dimers (7Ni) with a helical π-extended framework. Ultraviolet–Visible–Near-Infrared (UV–Vis–NIR) characterization revealed a split Soret band (446 and 562 nm) and a dramatically red-shifted Q-like band at 1262 nm (with a tail to 1400 nm) compared to the monomer (6). Another dimer (8Ni) displayed an even stronger absorption band at 1037 nm. Importantly, both 7Ni and 8Ni exhibited robust NIR-II PA signals, with clear peaks at 1083 nm and 1037 nm, respectively, confirming their utility as dual NIR-II absorption and PA imaging agents.

## Organic small-molecule NIR-II fluorophores for bioimaging

Optically mediated imaging technologies, characterized by their ability to directly visualize, map, and detect diverse biological information in living systems, have become powerful tools for both clinical diagnostics and basic biological research^72^. This field mainly includes two major branches: fluorescence imaging and PA imaging^201^. Among these, fluorescence imaging is preferred to traditional imaging modalities because of its advantages, such as low cost, high spatiotemporal resolution, and complete non-invasiveness, making it an ideal platform for real-time observation of biological processes^76^. The key to achieving superior fluorescence imaging performance lies in the rational selection and design of high-performance fluorescent probes. Among various fluorescent materials, organic fluorophores exhibit significant advantages over inorganic ones due to their excellent biocompatibility, flexible chemical modifiability, and tunable photophysical properties. Recent research has increasingly focused on endowing these organic materials with sophisticated functional characteristics, such as targeting specificity and stimuli-responsiveness, through precise molecular engineering strategies, including introducing targeting groups and constructing environment-responsive units^75^. This approach not only significantly enhances their imaging sensitivity and specificity but also substantially expands the dimensionality of information acquisition. Advanced imaging techniques based on these functionalized organic fluorophores show great potential for precise disease diagnosis and real-time therapeutic monitoring, drawing increasing interest for their future clinical translation.

### Cyanine-based fluorophores

**Fig. 14:**
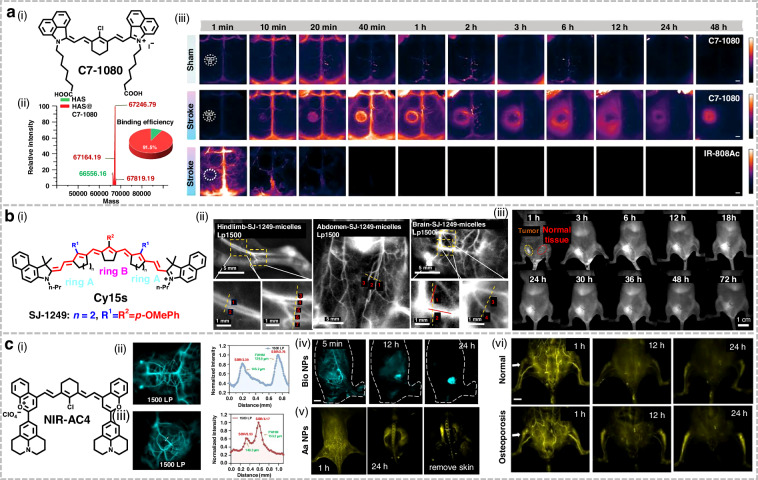
Structures and applications of cyanine-based NIR-II fluorophores in bioimaging. **a** i) Structure of C7-1080. ii) HRMS of the HSA@C7-1080 complex. iii) Dynamic imaging of sham/stroke mice following tail vein injection of C7-1080 or IR-808Ac. Reproduced with permission^107^. Copyright 2025, Wiley-VCH GmbH. **b** i) Structures of Cy15s. ii) NIR-IIb imaging of hindlimb, abdominal, and cerebral vessels in mice. iii) Abdominal NIR-II imaging of orthotopic 4T1 tumor-bearing mice following injection of SJ-1249-Micelles. Reproduced with permission^101^. Copyright 2025, Elsevier. **c** i) Structure of NIR-AC4. ii) Brain and iii) tumor vessel imaging of NIR-AC4 NPs in the NIR-II a/b windows. iv) The NIR-IIb intravital imaging of Bio-NPs at different time points post injection. v) The bone NIR-II imaging of Aa-NPs pre/post skin removal. vi) Real-time supine NIR-II fluorescence imaging of normal/osteoporotic mice post injection of Aa-NPs. Reproduced with permission^104^. Copyright 2025, Wiley-VCH GmbH

Direct in vivo visualization of albumin extravasation serves as a highly reliable strategy for assessing blood-brain barrier (BBB) disruption^202^. While traditional dyes like Evans Blue (EB) and ICG have been employed for BBB integrity evaluation, their limited albumin-binding specificity often leads to non-selective accumulation and significant adverse effects^203,204^. Zhu et al. developed an albumin-specific tagged probe, C7-1080, via a molecular side group engineering strategy (Fig. 14a)^107^. C7-1080 exhibited superior fluorescence brightness and photostability compared to ICG. In the photothrombotic stroke (PTS) model, the probe enabled high-sensitivity detection of BBB damage within just 10 min post injection, with results showing strong correlation with both 2,3,5-triphenyltetrazolium chloride (TTC) and EB staining. When paired with the albumin-escaping probe IR-800Ac, C7-1080 enabled real-time dual-channel imaging of BBB leakage and cerebral vessels. In general, in situ albumin-specific labeling showed great potential for stroke diagnosis and monitoring, and was expected to solve the important defects of current NIR-II fluorescent proteins.

Indocyanine fluorophores rank among the most clinically promising optical probes, yet achieving emission beyond 1,200 nm while retaining high brightness remains a significant challenge^205^. Zhang et al. first reported the design of novel indocyanine polymethine fluorophores (Cy15s), achieving record-breaking emission wavelengths beyond 1200 nm (Fig. 14b)^101^. In murine models, SJ-1271-Micelles facilitated high-contrast NIR-IIb vascular imaging, with SNRs of 6.79 (hindlimb), 3.39 (abdomen), and 5.87 (brain). Following intravenous injection, SJ-1271-Micelles enabled clear discrimination between tumors and adjacent normal tissues in orthotopic 4T1 tumor-bearing mice, with tumor-to-normal tissue ratio (TNR) and SNR peaking at 3.0 and 4.76, respectively. Remarkably, tumor-associated vasculature remained clearly detectable 72 h post injection, underscoring the significant potential of SJ-1271 for long-term tumor monitoring and vascular localization.

Soon afterward, Zhang et al. developed the novel heptamethine cyanine dye (NIR-AC4) through donor ectopic substitution at the terminal structure of NIR-II cyanine scaffold (Fig. 14c)^104^. Due to its stable NIR-II emission (1206 nm) and fluorescence quantum yield (QY = 0.063%), NIR-AC4 generated strong fluorescence signals in cerebral and tumor blood vessels. Biotin-modified and alendronate-functionalized NIR-AC4 NPs exhibited enhanced affinity for tumor and bone tissues, respectively. At 24 h post injection, NIR-AC4 Bio NPs attained a TNR of 15, affording high contrast NIR-IIb imaging for precise tumor resection and boundary delineation. Similarly, NIR-AC4 AaNPs yielded an SBR of 14.32, facilitating differentiation between spinous processes and vertebral bodies with enhanced structural integrity for precise osteoporosis detection in murine models.

**Fig. 15:**
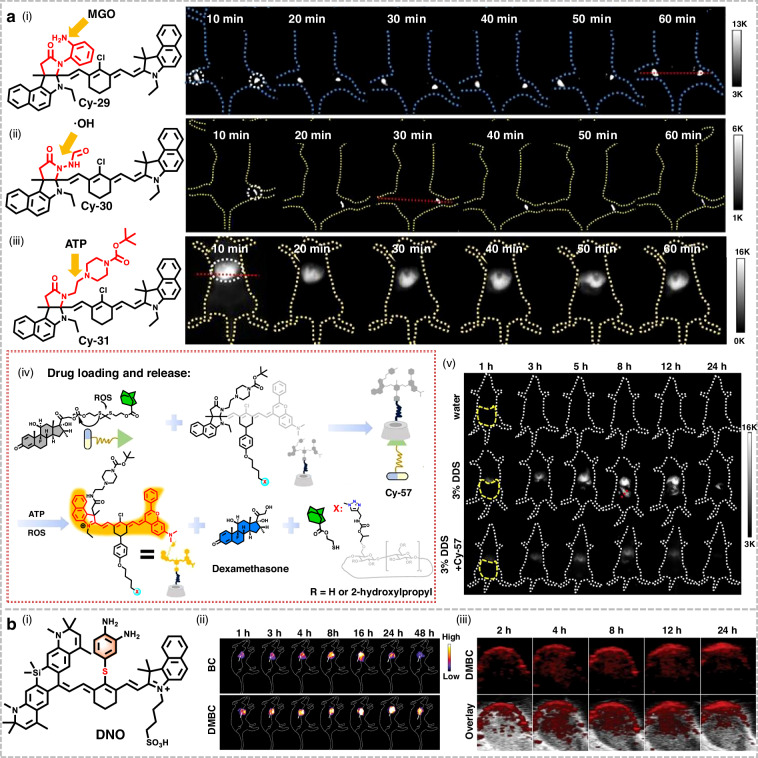
Structures and applications of activatable NIR-II cyanine probes in imaging. **a** i–iii) Structure of cyanine probes (Cy-29, Cy-30, and Cy-31) and corresponding time-dependent NIR-II fluorescence images post intratumor injection. iv) Construction of ATP/ROS dual-responsive theranostic probe Cy-57 and drug release process. v) Time-dependent NIR-II fluorescence imaging across experimental groups. Reproduced with permission^206^. Copyright 2025, Wiley-VCH GmbH. **b** i) Structure of DNO. ii) NIR-II imaging of Balb/c mice bearing subcutaneous tumors following intravenous injection of DNO NPs under 808 nm laser excitation. (BC the breast cancer group, DMBC the diabetic BC group) iii) Time-dependent photoacoustic images of DMBC mice following injection of DNO NPs. Reproduced with permission^108^. Copyright 2025, Wiley-VCH GmbH

Conventional heptamethine cyanine dyes exhibit persistent “always-on” fluorescence, leading to diminished imaging contrast and nonspecific responses in vivo. To overcome this limitation, Xiong et al. rationally engineered three activatable cyanine probes (Cy-29, Cy-30, and Cy-31) featuring diverse reactive groups (Fig. 15a)^206^. Upon activation by the target biomarkers, including methylglyoxal (MGO), hydroxyl radicals (•OH), and adenosine triphosphate (ATP), these probes showed 109–137-fold fluorescence enhancement at 940 nm, yielding high SBRs of 28:1, 13:1, and 38:1, respectively. Furthermore, leveraging host-guest chemistry, the researchers developed a dual-responsive NIR-II theranostic probe (Cy-57) capable of simultaneous sensing of ATP and ROS. It achieved highly sensitive ATP monitoring in inflammatory bowel disease (IBD) with an SBR of 48:1. This capability enabled precise therapeutic intervention and non-invasive detection of extracellular ATP in fecal samples. Collectively, these probes demonstrated excellent performance across multiple live models, highlighting their significant potential for advancing disease diagnosis and multiplexed detection of biomarkers in complex biological environments.

Breast cancer patients with diabetes exhibit significantly poorer survival outcomes than non-diabetic patients, though the underlying mechanisms remain unclear^207^. Li et al. developed a nitric oxide (NO)-activatable NIR-II fluorescent/PA dual-modal sensor, DNO (Fig. 15b)^108^. It operated via an intramolecular photoinduced electron transfer (PET) mechanism, enabling an “off/on” fluorescence switch. This design combined the advantages of fluorescence and PA imaging, including low background, high sensitivity, and deep tissue penetration, thereby surpassing single-modal approaches. In the diabetes-related breast cancer model, DNO NPs successfully enabled the detection of intratumoral NO expression levels, confirming that hyperglycemia induced by diabetes directly elevated NO concentrations within the tumor microenvironment (TME).

### BODIPY-based fluorophores

**Fig. 16:**
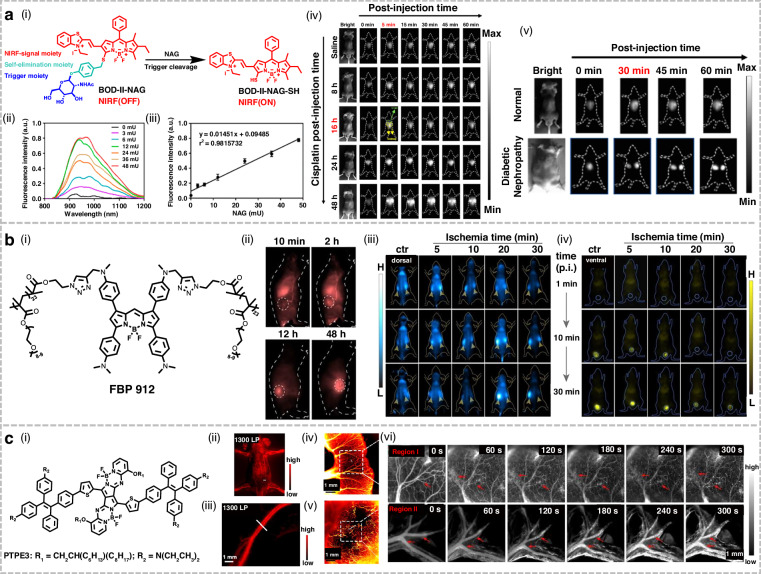
Structures and applications of BODIPY-based NIR-II fluorophores in imaging. **a** i) The mechanism of NAG detection by BOD-II-NAG-NP in vivo. ii) Fluorescence variation curve of BOD-II-NAG with increasing concentration of NAG. iii) The linear relationship between the fluorescence intensity changes and NAG concentration. iv) NIR-II fluorescence imaging of mice following intravenous injection of BOD-II-NAG-NP. v) NIR-II fluorescence imaging of mice with/without diabetic nephropathy following BOD-II-NAG-NP injection. Reproduced with permission^209^. Copyright 2021, American Chemical Society. **b** i) Structure of FBP 912. ii) Time-dependent bioimaging of 4T1-tumour-bearing mice following intravenous injection of FBP 912. iii, iv) In vivo NIR-II imaging (dorsal and ventral views) of renal ischemia-reperfusion mice at different time intervals post-ischemia treatment. Reproduced with permission^56^. Copyright 2021, Wiley-VCH GmbH. **c** i) Structure of PTPE3. In vivo NIR-II imaging of ii) whole-body blood vessels, iii) hindlimb vessels, iv) mesenteric micro-circulation and v) tumor vascular system following intravenous injection of PTPE3 NPs. vi) Functional and morphological changes in mesenteric microcirculation visualized by NIR-II fluorescence imaging in the V-PDT process. Reproduced with permission^160^. Copyright 2023, Elsevier

Conventional urinary enzyme assays lack sufficient sensitivity and exhibit high background interference for early diagnosis of acute kidney injury (AKI) or chronic kidney disease (CKD)^208^. Gu et al. developed BOD-II-NAG, a novel NIR-II fluorescent probe that could be activated by N-acetyl-β-D-glucosaminidase (NAG) (Fig. 16a)^209^. Fluorescence titration revealed that the addition of NAG led to a significant enhancement of its fluorescence intensity at 1000 nm, with a detection limit of 0.72 mU/mL. To improve biocompatibility, BOD-II-NAG was conjugated with mPEG-DSPE to form water-dispersible BOD-II-NAG-NP. In cisplatin-induced AKI models, BOD-II-NAG-NP enabled high-resolution imaging within 5 min post injection, with signals localized predominantly in the kidneys. Notably, in the high-fat diet-induced diabetic nephropathy model, the NIR-II fluorescence generated by BOD-II-NAG-NP could penetrate through the thicker fat layer, achieving clear imaging. These results indicated that in vivo NIR-II imaging of NAG held clinical application prospects for achieving early and accurate diagnosis of AKI and CKD.

Existing NIR-II organic renal-clearable probes generally suffer from insufficient brightness and short half-lives, which limit their further application in the high-resolution diagnosis of kidney disease^210^. Zhang et al. successfully developed a NIR-II brush fluorophore, FBP 912 (Fig. 16b)^56^. Its brush macromolecular structure effectively shielded the fluorophore core from quenching by water, endowing it with ~10-fold higher brightness than previously reported NIR-II renal-clearable organic probes. The high molecular weight conferred FBP 912 with a prolonged blood circulation time (*t*_1/2_ ≈ 6.1 h) and enhanced tumor accumulation, yielding an SNR of 8.2 in 4T1 tumor imaging. Furthermore, pharmacokinetic studies revealed that FBP 912 achieved 65% renal clearance at 12 h post injection, enabling real-time in vivo monitoring of renal ischemia-reperfusion injury. This design strategy might provide feasibility for diverse functional modifications and hold promise for broad applicability in designing water-soluble NIR-II probes.

Real-time monitoring of deep vasculature during vascular-targeted photodynamic therapy (V-PDT) is critically essential for evaluating therapeutic efficacy. Wang et al. pioneered the development of the NIR-II AIE fluorophore, PTPE3, specifically for dynamic fluorescence imaging of vascular dysfunction beyond 1300 nm during V-PDT (Fig. 16c)^160^. PTPE3 NPs, formed through assembly with Pluronic F127, exhibited exceptional optical properties, deep imaging penetration depth, and ultrahigh photochemical stability. Leveraging these advantages, PTPE3 NPs were applied to high-resolution NIR-II fluorescence imaging of vasculature across multiple sites in mice, including the abdomen, hindlimbs, mesenteric micro-circulation, and tumor. Furthermore, the NPs facilitated the real-time monitoring of mesenteric microcirculation responses and tumor vascular disruption throughout the V-PDT process, thereby promoting the development of advanced fluorescent bioprobes for bioimaging applications.

### BBTD-based fluorophores

**Fig. 17:**
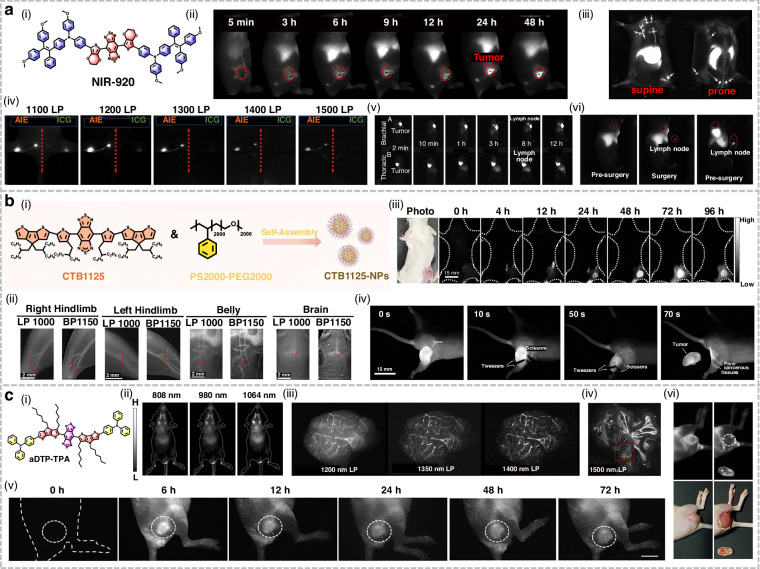
Structures and applications of BBTD-based NIR-II fluorophores in imaging. **a** i) Structure of NIR-920. ii) NIR-II tumor imaging of 4T1 tumor-bearing mice post intravenous injection of NIR-920 NPs. iii) NIR-II imaging of whole-body lymph node distribution. iv) Images of Left and right leg lymph node using NIR-920 NPs or ICG, respectively, with varying long-pass (LP) filters. v) NIR-II imaging revealed signal accumulation of NIR-920 NPs in the SLN of the 4T1 tumor. vi) Pre- and post-operative lymph node excision imaging. Reproduced with permission^126^. Copyright 2022, Elsevier. **b** i) The general procedure for preparation of CTB1125-NPs. ii) NIR-II imaging of specific body regions (right hindlimb, left hindlimb, belly, and brain) in mice post intravenous injection of CTB1125-NPs. iii) Time-dependent NIR-II imaging in mice post CTB1125-NPs injection. iv) NIR-II fluorescence image-guided H22 tumor resection. Reproduced with permission^141^. Copyright 2024, Wiley-VCH GmbH. **c** i) Structure of aDTP-TPA. ii) In vivo angiography under different excitation wavelengths. NIR-II imaging of iii) brain vessels and iv) mesenteric blood vessels. v) Tumor accumulation and imaging post intravenous injection of aDTP-TPA NPs. vi) NIR-II fluorescence image-guided 4T1 tumor resection. Reproduced with permission^215^. Copyright 2025, Wiley-VCH GmbH

Clinical sentinel lymph node (SLN) tracers, such as the radioisotope ^99m^Tc and ICG, suffer from limitations including short half-life, low resolution, and limited tissue penetration depth, making it urgent to explore advanced SLN tracers^211,212^. Wang et al. successfully developed a NIR-II AIE probe (NIR-920) via atomic substitution, for bioimaging and surgical navigation (Fig. 17a)^126^. Notably, NIR-920 exhibited a maximum absorption at 920 nm, which was red-shifted compared to previously reported aggregation-induced emission luminogens (AIEgens), and its emission extended beyond 1600 nm. NIR-920 NPs encapsulated by DSPE-mPEG2000 exhibited bright fluorescent properties, enabling high-resolution bioimaging of blood vessel, bone, tumor, and lymph node. Compared with ICG, NIR-920 NPs possessed advantages such as longer emission wavelength, higher SNR, and better photostability, and could be applied to long-term monitoring and tracking of deep tissues. In the 4T1 transplanted tumor mouse model, NIR-920 NPs achieved maximum accumulation in the brachial and thoracic lymph nodes 8 h after intratumoral injection. Benefiting from this distinct lymphnode enrichment capability, NIR-920 NPs enabled precise lymph node localization and facilitated NIR-II fluorescence imaging-guided lymph node resection.

The contradictory properties between emission wavelength and quantum yield of organic fluorophores typically result in significantly diminished brightness for fluorophores with emission >1100 nm^85,213^. This limitation frequently necessitates compensatory strategies such as prolonged exposure time and elevated laser power. Tang et al. developed a novel D-A-D type NIR-II fluorescent probe, CTB1125, which successfully achieved a balance between emission wavelength and quantum yield (Fig. 17b)^141^. In hexane solution, CTB1125 exhibited a peak emission at 1125 nm, with an exceptionally high quantum yield of 10.2%, surpassing all previously reported fluorophores with exceeding 1100 nm emission wavelength. Encapsulation of CTB1125 within amphiphilic PS2000-PEG2000 yielded CTB1125-NPs, retaining a substantial quantum yield of 4.84% in water. Compared with ICG, CTB1125-NPs exhibited superior tissue penetration depth and imaging SBR, enabling the clear delineation of vasculature in multiple regions of mice. Furthermore, CTB1125-NPs precisely delineated the boundary between tumor and normal tissue through NIR-II imaging under conditions of low dosage, minimal laser power, and short exposure time, thereby guiding the precise resection of H22 tumors. This study provided a high-performance NIR-II contrast agent for fluorescence imaging-guided surgery, holding considerable promise for clinical translation.

Organic AIEgens offer significant advantages for biomedical imaging, owing to their tunable spectral responses and high brightness^214^. Tang et al. developed a novel NIR-II excitable AIEgen, aDTP-TPA, for tumor resection guidance (Fig. 17c)^215^. It exhibited a broad absorption spectrum extending up to 1100 nm, with a maximum absorption coefficient of 9.14 × 10^3^ M^−1^ cm^−1^. Upon excitation at 980 nm, its emission spectrum extended to 1650 nm. Encapsulation of the AIEgen in DSPE-PEG2000 yielded water-dispersible aDTP-TPA NPs that demonstrated excellent tissue penetration depth, enabling high-resolution imaging of deep murine vasculature under 1064 nm excitation. Furthermore, the NPs exhibited outstanding performance in systemic angiography, as well as visualization of mesenteric and cerebral blood vessels. The Arg-Gly-Asp (cRGD)-functionalized aDTP-TPA NPs exhibited high tumor accumulation, enabling precise delineation of tumor boundaries via NIR-II imaging and thus effectively guiding the resection of 4T1 tumors. The numerous advantages of aDTP-TPA NPs open up new avenues for NIR-II bioimaging and therapeutic applications.

### Xanthene-based fluorophores

**Fig. 18:**
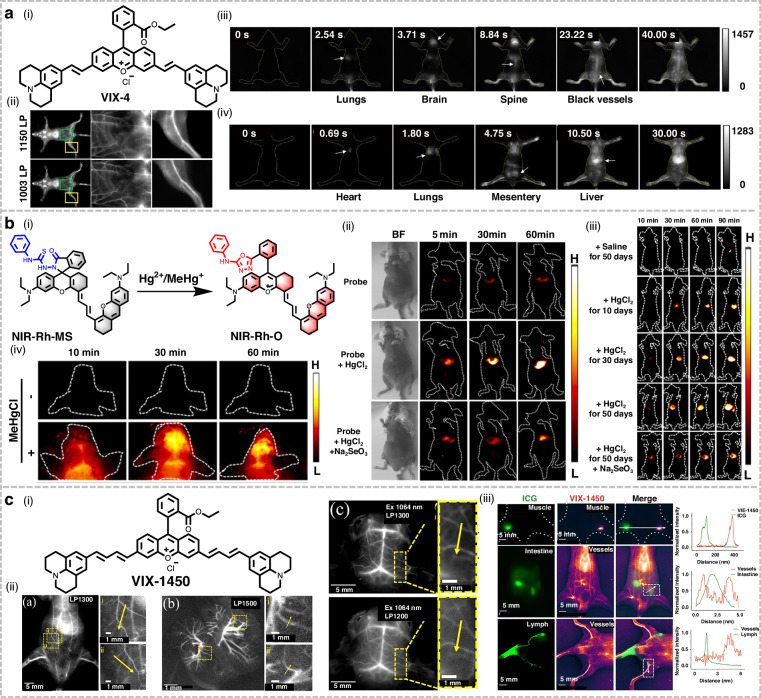
Structures and applications of xanthene-based NIR-II fluorophores in imaging. **a** i) Structure of VIX-4. ii) NIR-II fluorescence imaging of BALB/c mice administered VIX-4 liposomes. NIR-II fluorescence imaging from iii) ventral and iv) dorsal view at representative time points post injection of VIX-4. Reproduced with permission^170^. Copyright 2021, American Chemical Society. **b** i) Response mechanism of NIR-Rh-MS to Hg^2+^/MeHg^+^. NIR-II fluorescence imaging of mice with ii) acute and iii) chronic mercury poisoning at various time points following intravenous injection of NIR-Rh-MS. iv) NIR-II fluorescence cerebral imaging of mice with MeHgCl-induced mercury poisoning following intravenous injection of NIR-Rh-MS. Reproduced with permission^174^. Copyright 2022, Elsevier. **c** i) Structure of VIX-1450. ii) In vivo imaging of the entire vascular system, mesenteric vasculature, intestinal wall blood vessels and cerebral vessels in Balb/c nude mice following injection of VIX-1450 micelles. iii) Dual-channel imaging of ICG (left leg) and VIX-1450 micelles (right leg). Green channel (ICG): abdominal intestine and vasculature; purple-hot channel (VIX-1450): ventral lymphatic system and vasculature. Reproduced with permission^175^. Copyright 2025, American Chemical Society

Ma et al. developed a novel xanthene-based NIR-II dye, VIX-4, for dynamic imaging of blood circulation in living organisms (Fig. 18a)^170^. VIX-4 exhibited a maximum fluorescence emission at 1210 nm and high brightness in dichloromethane solution. Leveraging its exceptional optical properties, VIX-4 enabled clear visualization of abdominal and femoral vessels in mice. Furthermore, VIX-4 was employed to monitor the blood circulation system in mice, achieving high-speed dynamic imaging at 200 fps. Based on such superior spatiotemporal resolution, artery and vein could be directly distinguished through the direction of blood flow, and blood flow volume could be calculated. This study provided a novel scaffold for the development of dynamic imaging tools with high spatiotemporal resolution.

Accurate detection of inorganic mercury (Hg^2+^) and methylmercury (MeHg^+^) in vivo is crucial for understanding their toxicity and developing therapeutic interventions. However, existing methods suffer from limitations such as low imaging SBR, shallow penetration depth, and inability to simultaneously detect these two mercury species^216^. Liu et al. designed and synthesized a novel activatable NIR-II probe, NIR-Rh-MS, based on the xanthene “spirolactam close/open-switched fluorescence” strategy (Fig. 18b)^174^. This probe specifically responded to both Hg^2+^ and MeHg^+^, exhibiting concentration-dependent fluorescence enhancement at 1015 nm with detection limits of 40 nM and 1.14 μM, respectively. In acute and chronic mercury poisoning mouse models, NIR-Rh-MS enabled real-time monitoring of Hg^2+^-induced toxicity in the liver. Notably, NIR-Rh-MS successfully traversed the BBB, achieving for the first time the tracking of MeHg^+^ accumulation in the mouse brain. This breakthrough provided a potential approach for the real-time, high-precision monitoring of mercury poisoning across different organs.

In general, increasing the number of conjugated double bonds tends to lead to fragile chemical structures and poor photostability, as exemplified by cyanine dyes^217^. Cui et al. successfully synthesized a novel long-wavelength NIR-II dye, VIX-1450, through extension of the π-conjugation system, for application in fluorescence angiography (Fig. 18c)^175^. VIX-1450 exhibited a maximum emission peak at 1450 nm, and maintained exceptional chemical stability and photostability even at this extended wavelength. Benefiting from its large Stokes shift and long wavelength, VIX-1450 micelles were applied to high SNR imaging of the systemic, intestinal, and cerebral vasculature in mice. Furthermore, when employed in dual-color imaging with ICG, VIX-1450 demonstrated nearly negligible optical crosstalk. This facilitated precise labeling of distinct organs and allowed for more accurate assessment of physiological processes such as vascular permeability and lymphatic drainage.

### Cyano-based fluorophores

**Fig. 19:**
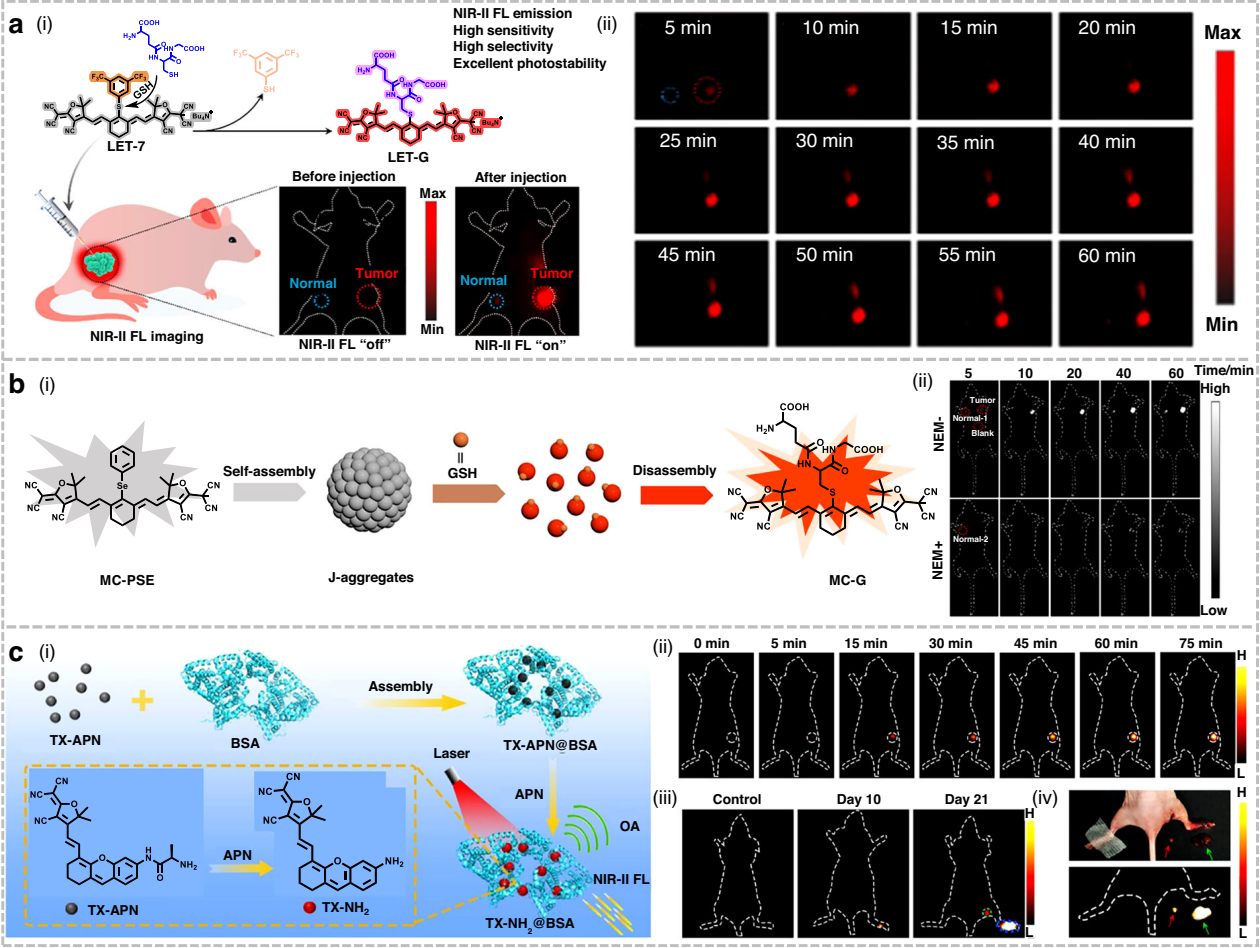
Structures and applications of cyano derivatives-based NIR-II fluorophores in imaging. **a** i) Schematic illustration of LET-7 for visualizing GSH in vivo. ii) Time-dependent NIR-II fluorescence imaging of mice following intratumoral (red circle) and subcutaneous (blue circle) injection of LET-7. Reproduced with permission^220^. Copyright 2021, American Chemical Society. **b** i) Structure of MC-PSE and mechanism of GSH detection. ii) Time-dependent NIR-II fluorescence imaging of mice with/without preinjection of NEM (N-ethylmaleimide, a GSH scavenger). Reproduced with permission^221^. Copyright 2023, American Chemical Society. **c** i) Schematic illustration for the construction of nanoprobe TX-APN@BSA and the mechanism for responding to APNs. ii) Time-dependent NIR-II fluorescence imaging of tumor-bearing mice following intratumoral injection of TX-APN@BSA. iii) NIR-II fluorescence imaging of model mice on days 10 and 21 post injection of 4T1 cells into the footpad. iv) Photograph and corresponding NIR-II fluorescence image of the model mice post 2nd tumor resection surgery. Reproduced with permission^223^. Copyright 2022, American Chemical Society

As a key endogenous non-protein biological thiol, GSH plays a pivotal role in maintaining intracellular redox balance^218^. Thus, the visualization of GSH is crucial for understanding its related pathophysiological processes. Existing fluorescent probes for GSH primarily operate within the visible and NIR-I regions, and their applications are limited by strong biological background interference and low tissue penetration^219^. Lin et al. pioneered the development of a GSH-activatable NIR-II probe (LET-7), enabling highly sensitive and selective visualization of GSH in vivo (Fig. 19a)^220^. Upon exposure to GSH, LET-7 exhibited a significant fluorescence enhancement at 928 nm, achieving a remarkably low detection limit of 85 nM. Remarkably, even 60 min post injection, LET-7 delineated the location of 4T1 tumors with high SBR and pronounced GSH selectivity. This study established a pioneering design paradigm for activatable NIR-II fluorescent probes targeting GSH detection.

Subsequently, Liu et al. developed a GSH-responsive NIR-II fluorescence/PA dual-mode probe (MC-PSE) (Fig. 19b)^221^. It tended to form stable J-aggregates in aqueous solution, and the presence of GSH triggered the disassembly of these J-aggregates, leading to the enhancement of fluorescent signals at 940 nm and the attenuation of PA signals at 980 nm. In the HCT-116 tumor-bearing mouse model, MC-PSE successfully achieved the visual detection of intracellular GSH in tumors, and accurately distinguished tumor tissue from normal tissue through NIR-II fluorescence/PA dual-modal imaging. Capitalizing on its unique mechanism of J-aggregate formation and disassembly, MC-PSE provided an effective platform for the accurate detection of GSH.

Lymphatic metastasis is a key mechanism by which cancer cells detach from the primary tumor, migrate to adjacent regional lymph nodes, and eventually spread to other organs or body sites^222^. Therefore, tracking lymphatic metastasis and performing image-guided tumor resection are of great significance. Wu et al. pioneered the development of an aminopeptidase N (APN)-activated PA/NIR-II fluorescence dual-modal imaging probe (TX-APN) (Fig. 19c)^223^. It was loaded into a bovine serum albumin (BSA) matrix to prepare the nanoprobe TX-APN@BSA, aiming to improve biocompatibility. This nanoprobe could be specifically activated by APN, generating strong fluorescent and PA signals at 922 nm and 795 nm, respectively. In the 4T1 tumor-bearing mouse model, TX-APN@BSA demonstrated efficacy in detecting and monitoring lymphatic metastasis through PA and NIR-II fluorescence imaging. Notably, guided by NIR-II fluorescence imaging, TX-APN@BSA was successfully applied for the intraoperative navigation of primary tumor and metastatic lymph node resection. This nanoprobe with excellent biosafety provided an important reference for the design of dual-modal probes targeting other biomarkers.

## Organic small-molecule NIR-II fluorophores for phototherapy applications

Multifunctional phototherapeutic technologies, integrating PDT, PTT, and diagnostic imaging capabilities, have garnered substantial and growing attention in biomedicine in recent years^76^. This interest stems from their non-invasive nature, high spatiotemporal precision, and exceptional light-controllable characteristics. The core mechanism of PDT relies on photosensitizers (PSs)^6,224^. Upon irradiation at a specific wavelength, PSs are excited from the ground state (S_0_) to the singlet excited state (S_1_), followed by efficient intersystem crossing (ISC) to the longer-lived triplet state (T_1_). The photoactivated PSs subsequently undergo Type I (electron transfer) and/or Type II (energy transfer to molecular oxygen) photochemical reactions, generating various highly cytotoxic ROS, including singlet oxygen (^1^O_2_), hydroxyl radicals (•OH), and superoxide anions (O_2_^−•^)^225^. These ROS induce apoptosis or necrosis in target cells, damage tumor vasculature, and may activate immune responses, collectively contributing to antitumor effects^26^.

However, the practical efficacy of PDT is crucially dependent on the performance of the employed PS. Current PDT faces several critical challenges, including insufficient tumor cell-killing potency, poor aqueous solubility of drugs, low photostability, and reduced ROS generation caused by aggregation under physiological conditions. These limitations mainly arise from the intrinsic molecular properties of the PSs. Moreover, most existing PSs are limited by excitation in tissue penetration-restricted regions, low ROS yields, and the absence of real-time therapeutic monitoring^226^. Therefore, screening and rational molecular design of high-performance PSs are central to improving PDT efficacy.

Distinct from the oxygen-dependent nature of PDT, PTT is an oxygen-independent therapeutic modality. During PTT, exogenous photothermal conversion agents absorb light energy and efficiently convert photon energy into heat primarily through non-radiative relaxation pathways, particularly internal conversion (IC). This process rapidly increases local temperature. The resultant controlled hyperthermia causes irreversible damage to tumor cells, such as protein denaturation and cell membrane rupture, ultimately leading to cell death. Notably, the heat signals generated during PTT can be captured by photothermal imaging (PTI) systems, providing high temperature sensitivity and real-time monitoring capability. Owing to its minimally invasive nature and favorable biosafety profile, PTT has been extensively investigated as a standalone tumor treatment strategy. Furthermore, hyperthermia (HT), although rarely used as a primary treatment, can enhance the selectivity and efficacy of chemotherapy or radiotherapy by inducing heat shock protein (HSP) expression and modulating the TME^226^.

However, the efficacy of monotherapies is often restricted by the complex TME. For instance, PDT is limited by tumor hypoxia, while PTT is constrained by the induction of thermotolerance through HSP expression. Consequently, the integration of PDT and PTT is recognized as a promising strategy to overcome the limitations imposed by the TME, effectively addressing their respective drawbacks and achieving synergistic therapeutic effects with improved overall outcomes. PTT-induced localized heating can promote tumor vascular dilation and increased blood perfusion, improving tissue oxygenation levels and thereby potentiating PDT efficacy. Conversely, PDT-induced cellular damage can compromise tumor cell thermotolerance, enhancing PTT-induced cytotoxicity. Therefore, developing integrated PTAs that combine PDT and PTT is of great significance.

### Cyanine-based PTAs

**Fig. 20:**
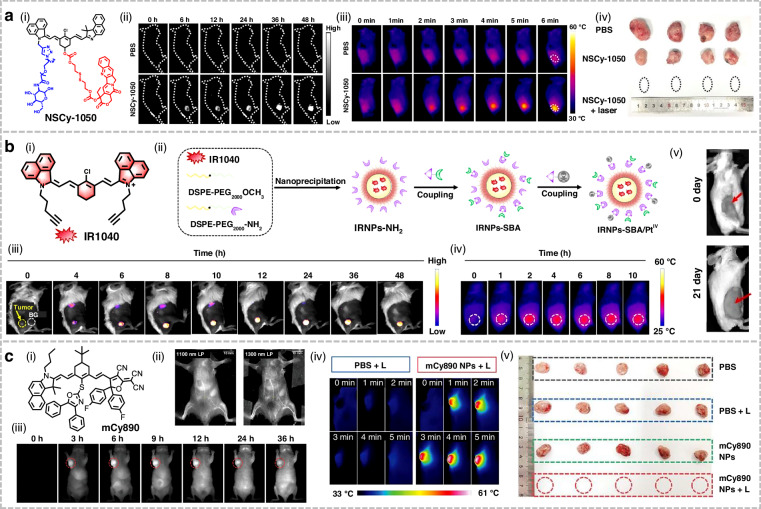
Structures and applications of cyanine-based NIR-II molecules in phototherapy. **a** i) Structure of NSCy-1050. ii) Time-dependent NIR-II fluorescence imaging of 4T1 tumor-bearing mice following intravenous injection of NSCy-1050. iii) Photothermal images of mice under different irradiation durations. iv) Representative tumor photographs from different treatment groups at day 20. Reproduced with permission^88^. Copyright 2023, Wiley-VCH GmbH. **b** i) Structure of IR1040. ii) Schematic illustration for the preparation of IRNPs-SBA/Pt^IV^. iii) Time-dependent NIR-II fluorescence imaging of Pan02 tumor-bearing mice following intravenous injection of IRNPs-SBA/Pt^IV^. iv) Infrared thermal images of mice at 24 h post intravenous injection of IRNPs-SBA/Pt^IV^ under different irradiation durations. v) Photographs of mice pre-treatment (day 0) and post-treatment (day 21). Reproduced with permission^95^. Copyright 2023, Elsevier. **c** i) Structure of mCy890. ii) Blood vessel imaging of mice injected with mCy890 NPs. iii) Time-dependent NIR-II fluorescence imaging of tumor-bearing mice following intravenous injection of mCy890 NPs. iv) Photothermal images of mice under different irradiation durations. v) Representative tumor photographs from different treatment groups at day 14. Reproduced with permission^93^. Copyright 2024, Wiley-VCH GmbH

Conventional NIR-II cyanine dyes often suffer from inadequate tumor targeting and poor aqueous solubility, leading to pronounced hepatic background fluorescence that complicates accurate diagnosis and treatment of extrahepatic diseases^66^. Xiong et al. developed a NIR-II theranostic agent (NSCy-1050) through covalent conjugation of a dual pH/viscosity-responsive fluorophore (NSCy-975) with water-soluble PEG-glucosamine and camptothecin (CPT) (Fig. 20a)^88^. The rational molecular design of NSCy-1050 enabled both glucose transporter 1 (GLUT1) targeting and GSH-activated self-immolative release of CPT. In 4T1 breast tumor models, NSCy-1050 demonstrated exceptional tumor-specific accumulation, achieving an unprecedented tumor-to-liver fluorescence ratio of 19.5:1. Upon 808 nm laser irradiation, the tumor temperature rapidly reached 56 °C within 6 min. The synergistic effect of PTT mediated by NSCy-1050 and chemotherapy via GSH-triggered CPT release resulted in potent tumor suppression. This work established a universal strategy for constructing “dual-locked” cyanine probes capable of integrating NIR-II fluorescence-guided in vivo imaging with therapeutic functions.

CAs, which are overexpressed in diverse tumor cells, serve as a pivotal regulator of hypoxic and acidic TME and have emerged as a critical target for cancer theranostics^227,228^. Ye et al. constructed a CA-targeted theranostic nanoprobe (IRNPs-SBA/Pt^IV^), for NIR-II fluorescence imaging-guided pancreatic cancer treatment (Fig. 20b)^95^. The theranostic nanoprobe achieved tumor-specific accumulation through its high binding affinity for CA (*K*_d_ = 14.40 ± 5.49 nM), enabling highly sensitive NIR-II imaging with a tumor-to-background ratio of 7.2. Elevated endogenous GSH levels triggered the reduction of Pt^IV^ to the active chemotherapeutic Pt^II^, while concomitant GSH depletion further upregulated intracellular ROS, amplifying the chemotherapeutic effect. Under the guidance of NIR-II imaging, low-power 1064 nm laser irradiation triggered a synergistic photothermal-chemotherapeutic effect, effectively suppressing pancreatic tumor growth in mice with minimal toxicity.

To improve the poor photostability and low PCE of ICG, Li et al. transformed ionic cyanines into neutral polymethine merocyanine (mCy890) (Fig. 20c)^93^. The water-soluble nanoparticles prepared by co-assembly of mCy890 with Pluronic F127 exhibited peak emission at 1034 nm and a remarkable PCE of 51%, markedly surpassing that of ICG. mCy890 NPs preferentially accumulated at tumor sites, reaching peak enrichment 6 h post-administration, enabling clear visualization of the vasculature in mice. Leveraging this targeted delivery, PTT mediated by mCy890 NPs achieved rapid 4T1 tumor temperature elevation to 60.5 °C within 5 min of laser irradiation (808 nm), resulting in potent tumor suppression with minimal off-target toxicity. This work constituted the first demonstration of biomedical-grade NIR-II merocyanines, offering a novel avenue for efficient tumor phototheranostics guided by neutral polymethine merocyanine dyes.

### BODIPY-based PTAs

**Fig. 21:**
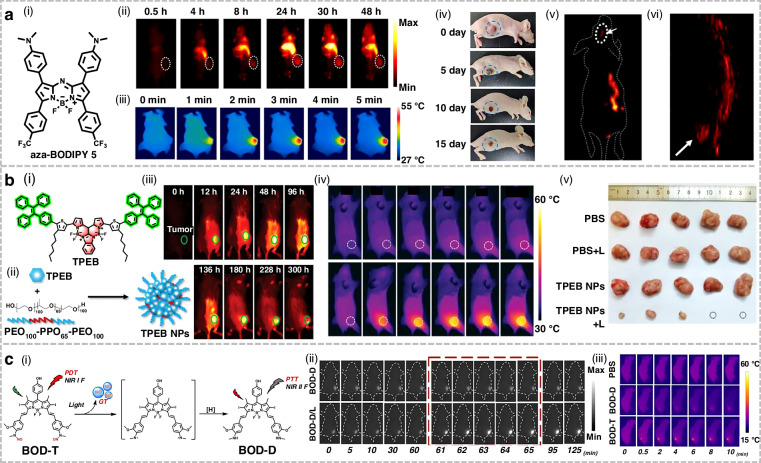
Structures and applications of BODIPY-based NIR-II molecules in phototherapy. **a** i) Structure of aza-BODIPY 5. ii) Time-dependent NIR-II fluorescence imaging of 4T1 tumor-bearing mice post intravenous injection of 5@FAP-NP. iii) IR thermal images of mice post intravenous injection of 5@FAP-NP under different irradiation durations. iv) Representative tumor photographs from different treatment groups of 4T1 tumor-bearing mice following PTT. v) NIR-II fluorescence and vi) photoacoustic imaging of GBM-bearing mice following injection of 5@FAP-NP. Reproduced with permission^164^. Copyright 2024, SCUT, AIEI, and John Wiley & Sons Australia, Ltd. **b** i) Structure of TPEB. ii) The general procedure for preparation of TPEB NPs. iii) Time-dependent NIR-II fluorescence imaging of tumor-bearing mice post intravenous injection of TPEB NPs. iv) Thermal images of mice post intravenous injection of TPEB NPs under different irradiation durations. v) Representative tumor photographs from different treatment groups at day 14. Reproduced with permission^166^. Copyright 2024, Wiley-VCH GmbH. **c** i) Mechanisms of light-triggered NO release and switching between imaging/phototherapy modes. ii) Time-dependent NIR-II fluorescence imaging of HeLa tumor-bearing mice with/without UV irradiation post subcutaneous injection of BOD-D. iii) Photothermal images of mice under different irradiation durations. Reproduced with permission^168^. Copyright 2025, SCUT, AIEI, and John Wiley & Sons Australia, Ltd

The development of integrated platforms for tumor targeting, multimodal real-time imaging, and efficient phototherapy holds significant necessity for tumor theranostics, particularly for deeply invasive aggressive malignancies such as glioblastoma (GBM). Zhang et al. reported a novel NIR-II chromophore (aza-BODIPY 5) for non-invasive PTT guided by NIR-II fluorescence/PA dual-modality imaging (Fig. 21a)^164^. To augment tumor and brain-targeting capability, amphiphilic block copolymers DSPE-PEG2000-FA were employed to encapsulate aza-BODIPY 5, yielding the water-dispersible 5@FAP-NP. In the 4T1 xenograft mice model, 5@FAP-NP administration resulted in intense NIR-II fluorescence and PA signals at the tumor site, indicative of substantial accumulation within tumor tissue. Upon 880 nm laser irradiation, 5@FAP-NP facilitated a rapid temperature increase at the tumor site to 49.7 °C within 5 min, enabling effective tumor ablation. Furthermore, pronounced NIR-II fluorescence and PA signals were detected in the brain tissue of GBM-bearing mice 24 h post injection of 5@FAP-NP, allowing clear visualization of the deep GBM in vivo and guidance for subsequent PTT. The development of 5@FAP-NP provided a valuable reference framework for the design of phototheranostic agents targeting brain tumors, demonstrating substantial potential. AIEgens have garnered extensive attention due to their inherent AIE properties and large Stokes shifts. However, their output performance in bioimaging and phototherapy is frequently constrained by unsatisfactory molar extinction coefficients^6^. Tang et al. constructed a novel AIEgen (TPEB) with excellent photon-harvesting capability (ε = 1.29 × 105 M^−1^ cm^−1^) through the “aggrandizing absorption reservoir” strategy (Fig. 21b)^166^. PEO100-PPO65-PEO100 (F127)-encapsulated TPEB NPs exhibited a high fluorescence quantum yield of 5.04%. In vivo imaging of 4T1 tumor-bearing mice revealed efficient accumulation of TPEB NPs at tumor sites, with a distinct NIR-II fluorescence signal observable 12 h post-administration. Capitalizing on the excellent light-capturing ability and fluorescence quantum yield, TPEB NPs were successfully employed for NIR-II imaging-guided PTT. Upon laser irradiation, the tumor temperature rapidly increased to 55.8 °C within 5 min, attaining a remarkable 94% tumor inhibition rate.

Most reported photosensitizers are confined to either PDT or PTT, fundamentally limited by hypoxic TME and heat shock response, respectively^229^. Qu et al. pioneered an intelligent and controllable photosensitizer (BOD-D) capable of light-triggered switching among PDT, gas therapy, and PTT modalities (Fig. 21c)^168^. Unactivated BOD-D exhibited bright NIR-I emission and potent ^1^O_2_ generation capability. To circumvent PDT efficacy attenuation from persistent oxygen depletion, UV irradiation was used to trigger rapid NO dissociation from BOD-D. The resulting transformation product, BOD-T, facilitated NIR-II fluorescence imaging-guided PTT, rapidly elevating tumor temperatures to ~50 °C within minutes. Notably, the released NO functioned as a gas therapeutic agent while sensitizing PDT and PTT, demonstrating synergistic therapeutic efficacy in cervical cancer treatment.

### BBTD-based PTAs

**Fig. 22:**
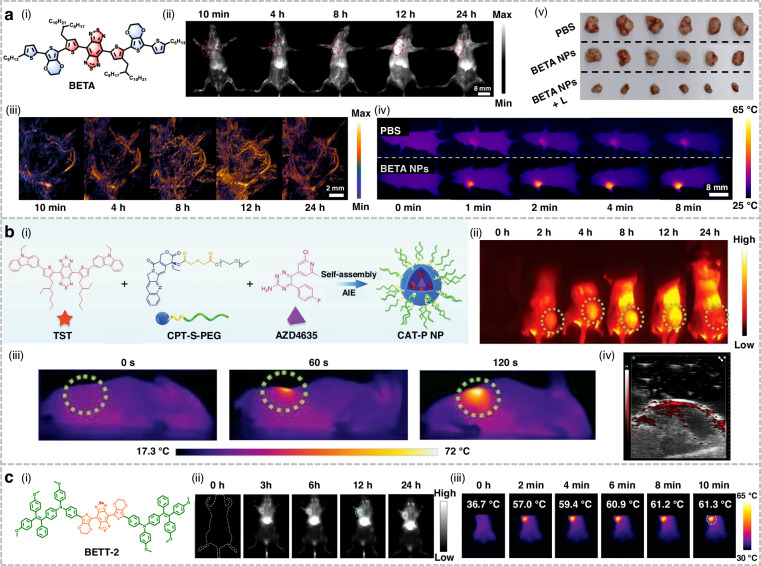
Structures and applications of BBTD-based NIR-II molecules in phototherapy for conventional tumors. **a** i) Structure of BETA. Time-dependent NIR-II ii) fluorescence and iii) photoacoustic imaging of 4T1 tumor-bearing mice following intravenous injection of BETA NPs. iv) Photothermal images of mice under different irradiation durations. v) Representative tumor photographs from different treatment groups of 4T1 tumor-bearing mice. Reproduced with permission. Reproduced with permission^129^. Copyright 2022, Wiley-VCH GmbH. **b** i) The general procedure for preparation of CAT-NP. ii) NIR-II fluorescence, iii) photothermal, and iv) photoacoustic images of CAT-NP in vivo. Reproduced with permission. Reproduced with permission^120^. Copyright 2022, Wiley-VCH GmbH. **c** i) Structure of BETT-2. ii) Time-dependent NIR-II fluorescence imaging of 4T1 tumor-bearing mice following intravenous injection of BETT-2 NPs. iii) Thermal images of mice and temperature changes of tumor sites at 12 h post injection of BETT-2 NPs under different irradiation durations. Reproduced with permission. Reproduced with permission^139^. Copyright 2024, Wiley-VCH GmbH

Researchers frequently employ the strategy of constructing large π-conjugated molecular skeletons with distorted molecular structures to extend the absorption range of D-A-D type conjugated small molecules, aiming to achieve intense NIR-II absorption and excellent phototheranostic performance. Regrettably, this strategy has not effectively addressed the issue of low molar extinction coefficient in the NIR-II region^223^. Fan et al. successfully developed a high-performance photosensitizer (BETA) for NIR-II fluorescence/PA imaging-guided PTT (Fig. 22a)^129^. The BETA NPs encapsulated by Pluronic F-127 exhibited a maximum absorption at 892 nm, with a high ɛ_1064_ of 1.13 × 10^4^ M^−1^ cm^−1^, achieving a maximum NIR-II PCE of 47.6%. Owing to the exceptional light-capturing capability at 1064 nm, high-resolution NIR-II fluorescence and PA imaging could be achieved using laser excitation at this specific wavelength. In xenograft 4T1 tumor-bearing mice, BETA NPs reached peak accumulation at the tumor site 12 h post intravenous injection, exhibiting intense NIR-II fluorescence and PA signals. Photothermal imaging revealed that BETA NPs elevated the tumor temperature to 62.8 °C under 1064 nm laser irradiation for 8 min, resulting in near-complete tumor elimination.

PTAs necessitate the integration of fluorescence imaging and PDT/PTT capabilities. However, achieving an optimal balance between these functions remains challenging, as they rely on distinct radiative and non-radiative decay pathways, respectively. Consequently, developing intelligent nanodrug delivery systems that dynamically regulate the decay pathways is imperative. Yu et al. successfully synthesized a novel AIE molecule (TST) with both NIR-II imaging and PTT capabilities (Fig. 22b)^127^. To further enhance therapeutic efficacy, camptothecin prodrug (CPT-S-PEG) and immune checkpoint inhibitor (AZD4635) were co-assembled with TST to form CAT-NP. Within intact nanoparticles, the strong CPT-TST interaction restricts the intramolecular rotation of TST, suppressing non-radiative decay and endowing CAT-NP with exceptional NIR-II imaging performance (emission: 1050 nm; quantum yields: 15.32%). Following cellular uptake by cancer cells, the redox-sensitive CPT-S-PEG underwent degradation triggered by H_2_O_2_. The released TST, freed from the CPT restriction, exhibited significantly enhanced non-radiative decay, thereby promoting efficient photothermal conversion. In 4T1 tumor-bearing mice, CAT-NP enabled high-resolution NIR-II fluorescence imaging and PTT, elevating tumor temperature to 72 °C upon irradiation for 2 min and significantly inhibiting tumor growth. Notably, PTT-induced immunogenic cell death (ICD) in cancer cells results in substantial ATP release. Released AZD4635 blocked the CD39-CD73-A2AR signaling axis, preventing the excessive conversion of ATP into immunosuppressive adenosine, thereby enhancing immunotherapy efficacy. This intelligent nano-delivery system achieved synergistic integration of NIR-II imaging-guided PTT, chemotherapy, and immunotherapy, offering a highly promising strategy for tumor theranostics.

Multimodal phototheranostic systems, which skillfully integrate diverse modalities, have garnered widespread attention among researchers. AIEgens are ideal candidates for constructing multimodal PTAs, thanks to their superiority in balancing various competitive energy dissipation pathways^6,230^. Wang et al. pioneered the development of BETT-2, a NIR-II-excitable AIEgen with multimodal phototheranostic properties (Fig. 22c)^139^. The maximum absorption and emission of BETT-2 reach 915 nm and 1240 nm, respectively, which are highly favorable for NIR-II fluorescence imaging. BETT-2 NPs prepared by encapsulation with DSPE-mPEG2000 exhibited a high molar absorptivity (1813.56 M^−1^ cm^−1^) at 1064 nm, coupled with excellent ROS generation capacity and PCE (56.6%). Capitalizing on these outstanding optical characteristics, BETT-2 NPs were applied to high-contrast NIR-II fluorescence/PA/photothermal trimodal imaging. In 4T1 tumor-bearing mice, BETT-2 NPs reached peak accumulation at the tumor site 12 h post injection, demonstrating intense NIR-II fluorescence and PA signals. Upon 1064 nm laser irradiation, BETT-2 NPs induced a rapid temperature elevation to 61.3 °C within 10 min at the tumor site, leading to the complete eradication of tumors by the 3rd day after treatment. The successful implementation of PDT/PTT based on BETT-2 provided an important theoretical basis for the development of NIR-II activated phototheranostic systems suitable for cancer treatment.

**Fig. 23:**
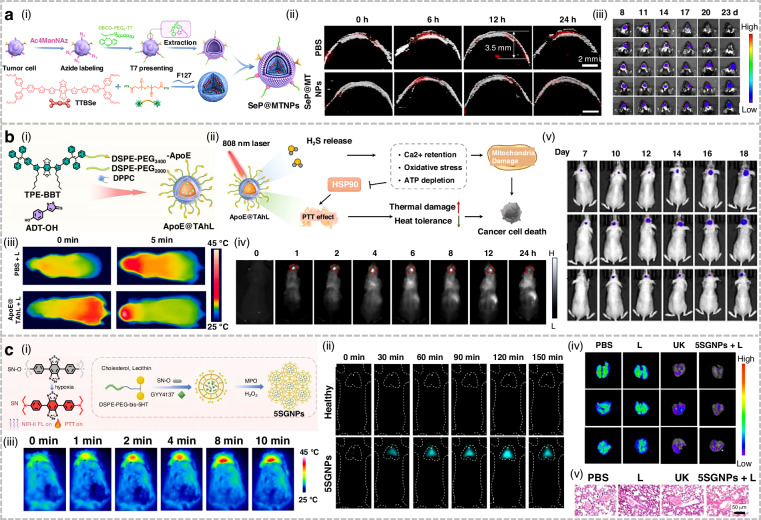
Structures and applications of BBTD-based NIR-II molecules in phototherapy for GBM and PE. **a** i) The general procedure for preparation of SeP@MT NPs. ii) Time-dependent photoacoustic/ultrasound merged images of GL261 tumor-bearing mice following injection of SeP@MT NPs. iii) Time-dependent bioluminescence images of orthotopic GBM following injection of SeP@MT NPs. Reproduced with permission^149^. Copyright 2025, Wiley-VCH GmbH. **b** i) The general procedure for preparation of ApoE@TAhL. ii) Mechanism of synergistic therapy of ApoE@TAhL against in situ GBM. iii) Thermal images of mice post intravenous injection of ApoE@TAhL upon 808 nm laser irradiation. iv) Time-dependent NIR-II fluorescence imaging of GBM-bearing mice following injection of ApoE@TAhL. v) Time-dependent bioluminescence images of orthotopic GBM following injection of ApoE@TAhL. Reproduced with permission^234^. Copyright 2025, Wiley-VCH GmbH. **c** i) The general procedure for preparation of 5SGNPs. ii) Time-dependent NIR-II fluorescence signal intensity in the lungs of PE mice following intravenous injection of 5SGNPs. iii) IR thermal images of 5SGNPs-treated PE mice under different irradiation durations. iv) Fluorescence signal intensity of FITC-fibrin in the lungs of mice after different treatments. v) Representative H&E-stained lung sections from different treatment groups. Reproduced with permission^236^. Copyright 2025, American Chemical Society

GBM poses significant challenges to clinical treatment due to its invasive nature, poor prognosis, and high recurrence rate^231^. PA imaging has emerged as a highly promising diagnostic modality for deep-seated brain tumors owing to its deep tissue penetration and low tissue scattering properties^232^. Qi et al. designed and synthesized a novel selenium-containing chromophore (TTBSe), which exhibits significant PCE and PA signal generation (Fig. 23a)^149^. After encapsulating TTBSe and the paclitaxel (PTX) prodrug PTX-azo with Pluronic F-127, the nanoparticles were coated with glioma cell membrane, and finally conjugated with the transferrin-mimicking peptide T7 on the membrane surface to prepare SeP@MT NPs. The T7 peptide endowed SeP@MT NPs with enhanced BBB penetration and glioma-targeting capabilities through specific binding to transferrin receptors overexpressed on both the BBB and GBM tumors. In GL261 tumor-bearing mice, SeP@MT NPs achieved maximum accumulation at the brain tumor site 12 h post injection, generating high-contrast NIR-II PA signals that enabled precise GBM localization and tumor margin delineation. Under 1064 nm laser irradiation, the photothermal effect generated by SeP@MT NPs combined with the heat-triggered activation of PTX-azo synergistically induced ICD, further enhancing tumor eradication efficacy and amplifying antitumor immune responses. This theranostic nanoplatform integrated NIR-II PA imaging, brain tumor targeting, and self-synergistic immunotherapy, establishing a novel paradigm for addressing the diagnostic and therapeutic challenges of immunologically “cold” tumors such as GBM.

Small-molecule gases exhibit high tissue penetration capability. Hydrogen sulfide (H_2_S)-based gas therapy is capable of inducing oxidative stress and downregulating ATP-dependent HSP90 synthesis, making its combination with PTT a promising approach to achieve synergistic therapeutic effects^233^. Tang et al. innovatively developed a NIR-II-emissive nanosystem for the synergistic treatment of GBM (Fig. 23b)^234^. Specifically, the NIR-II AIE photothermal agent TPE-BBT and H_2_S prodrug ADT-OH were co-encapsulated using DSPE-PEG and liposome DPPC, followed by surface functionalization with the brain-targeting ApoE peptide to prepare ApoE@TAhL. In vivo experiments showed that ApoE@TAhL effectively penetrated the BBB and accumulated at the GBM site, exhibiting superior NIR-II real-time imaging performance and photothermal effect under 808 nm laser irradiation. The photothermal effect further promoted the deformation of the temperature-sensitive liposomal membrane, triggering the release of H_2_S, which induced intracellular oxidative stress and downregulation of ATP levels. Reduced ATP levels led to decreased HSP90 expression, thereby suppressing tumor thermotolerance and enhancing PTT efficacy. This strategy of integrating non-invasive H_2_S gas “bomb” into GBM phototherapy offers a solution to the challenges of insufficient laser penetration depth and suboptimal photothermal therapeutic outcomes.

Pulmonary embolism (PE), a common and potentially fatal manifestation of venous thromboembolic disease, continues to present challenges for precise monitoring and therapeutic intervention^235^. Qi et al. developed a hypoxia-responsive NIR-II photosensitizer (SN-O) with an N-oxide structure (Fig. 23c)^236^. It was co-encapsulated with the thermosensitive H_2_S donor GYY4137 in DSPE-PEG functionalized with bis-tryptophan (bis-5HT) to prepare 5SGNPs. Notably, 5SGNPs tended to form large aggregates through the reaction between surface bis-5HT and myeloperoxidase (MPO), thereby achieving selective targeting to thrombus sites. Furthermore, 5SGNPs demonstrated hypoxia-induced photothermal effects (with PCE of 39%) and H_2_S release capability upon 808 nm laser irradiation. In PE mice, pulmonary accumulation of 5SGNPs peaked at 2 h post-administration, accompanied by strong NIR-II fluorescence signals with SNR of 124. Upon irradiation, 5SGNPs elevated the lung surface temperature to ~42 °C within 10 min. Compared to free urokinase (UK), this nanosystem achieved rapid and efficient thrombus degradation by combining PTT with H_2_S release. This multifunctional hypoxia-responsive platform was conducive to promoting the precise diagnosis and treatment of cardiovascular diseases such as PE.

### Xanthene-based PTAs

**Fig. 24:**
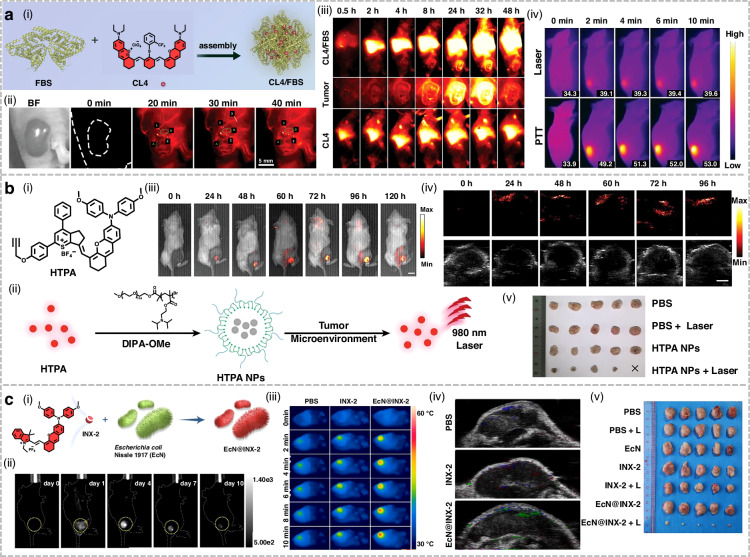
Structures and applications of xanthene-based NIR-II molecules in phototherapy. **a** i) The general procedure for preparation of CL4/FBS complex. Time-dependent NIR-II fluorescence imaging of ii) tumor vessels and iii) 4T1 tumor-bearing mice following intravenous injection of CL4/FBS. iv) IR thermal images of CL4/FBS-treated mice under different irradiation durations. Reproduced with permission^238^. Copyright 2022, Wiley-VCH GmbH. **b** i) Structure of HTPA. ii) The general procedure for preparation of HTPA NPs and release of HTPA. Time-dependent NIR-II iii) fluorescence and iv) photoacoustic images in subcutaneous 4T1-Luc models following intravenous injection of HTPA NPs. v) Representative tumor photographs from different treatment groups of 4T1 tumor-bearing mice. Reproduced with permission^239^. Copyright 2025, Wiley-VCH GmbH. **c** i) The general procedure for preparation of AIEgen bacteria hybrid bionic robot EcN@INX-2. ii) Time-dependent NIR-II fluorescence imaging of tumor-bearing mice following intravenous injection of EcN@INX-2. iii) Photothermal and iv) photoacoustic images of mouse tumor tissue at 24 h post injection of INX-2/EcN@INX-2. v) Representative tumor photographs from different treatment groups of CT26 tumor-bearing mice. Reproduced with permission^178^. Copyright 2025, Springer Nature

Binding with fetal bovine serum (FBS) may serve as an effective approach to enhance the hydrophilicity and fluorescence intensity of hydrophobic dyes^237^. Wang et al. synthesized a novel NIR-II xanthene dye (CL4) for tumor theranostics (Fig. 24a)^238^. In aqueous solution, the CL4/FBS complex displayed a blue-shifted maximum emission at 1235 nm, alongside a 3.6-fold fluorescence intensity enhancement and a PCE of 36%. Compared to CL4 alone, the complex showed significantly reduced cytotoxicity, indicating improved biocompatibility. Furthermore, this system demonstrated exceptional capability for NIR-II fluorescence imaging of tumor vasculature, achieving a resolution of 0.23 mm. Following intravenous injection, the complex attained peak tumor accumulation at 32 h, demonstrating significantly superior tumor enrichment efficacy relative to free CL4. Upon irradiation with 1064 nm laser, CL4/FBS complex elevated the tumor temperature to 53 °C within 10 min, effectively suppressing 4T1 tumor growth through PTT. This strategy of forming complexes with FBS provided a novel approach for balancing fluorescence intensity and PTT efficacy, holding significant promise for advancing theranostics of deep-seated tumors.

Stimulus-responsive probes demonstrate significant advantages in tumor detection and diagnosis due to their high imaging contrast and specificity. Xiao et al. successfully developed a pH-responsive NIR-II photosensitizer, HTPA (Fig. 24b)^239^. In aqueous environments, the aggregation process of HTPA, driven by twisted intramolecular charge transfer (TICT) and strong π-π stacking interactions, enhanced non-radiative transitions. This not only amplified the PA signal but also retained the NIR-II imaging capability. Notably, the photosensitizing properties of HTPA exhibited pH-dependent behavior, enabling reversible modulation in response to pH variations. “Off-state” HTPA NPs were prepared by encapsulating HTPA with DIPA-OMe to shield its absorption. In 4T1 tumor-bearing mouse models, HTPA NPs demonstrated significant accumulation at the tumor site, with fluorescence signals peaking at 96 h post injection. Under 1064 nm laser irradiation, HTPA NPs enabled high-contrast PA imaging, achieving an SNR of 96. Furthermore, HTPA NPs specifically targeted mitochondria in tumor cells and effectively suppressed tumor growth through PDT.

Limitations of traditional photosensitizers, such as constrained tumor penetration and retention as well as the requirement for multiple irradiation sessions, restrict their broader application. Li et al. developed a hybrid bionic robot (EcN@INX-2) by conjugating the multifunctional AIEgen (INX-2) with Escherichia coli Nissle 1917 (EcN) (Fig. 24c)^178^. The superior optical properties of INX-2 synergized with the inherent tumor-targeting capability of EcN, endowing the EcN@INX-2 with enhanced tumor tropism, multimodal imaging capacity, and synergistic therapeutic functions. In CT26 tumor-bearing mice, EcN@INX-2 selectively accumulated within the hypoxic tumor regions, enabling fluorescence/photothermal/PA trimodal imaging. Upon laser irradiation, EcN@INX-2 elevated the tumor temperature to 51 °C, effectively suppressing CT26 tumor growth through combined PDT/PTT/ICD mechanisms. This study, by integrating AIEgens with bacteria to construct a multifunctional bionic robot, provided a highly promising solution for imaging-guided targeted photoimmunotherapy of colon cancer.

### Cyano-based PTAs

**Fig. 25:**
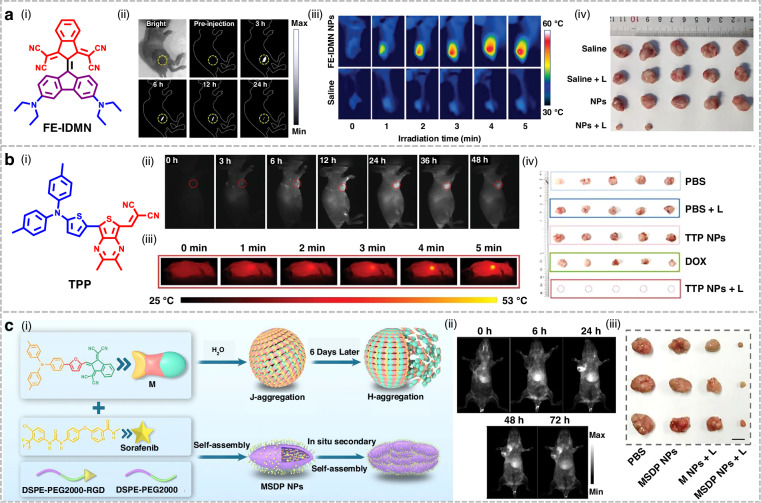
Structures and applications of cyano derivatives-based NIR-II molecules in phototherapy. **a** i) Structure of FE-IDMN. ii) Time-dependent NIR-II fluorescence imaging of 4T1 tumor-bearing mice following intratumoral injection of FE-IDMN NPs. iii) Photothermal images of mouse tumor tissue at 4 h post injection of FE-IDMN NPs under different irradiation durations. iv) Representative tumor photographs from different treatment groups at day 14. Reproduced with permission^187^. Copyright 2023, Wiley-VCH GmbH. **b** i) Structure of TTP. ii) Time-dependent NIR-II fluorescence imaging of MCF-7 tumor-bearing mice following injection of TTP NPs. iii) Infrared thermal images of mouse tumor tissue post injection of TTP NPs under different irradiation durations. iv) Representative tumor photographs from different treatment groups of MCF-7 tumor-bearing mice. Reproduced with permission^188^. Copyright 2024, Wiley-VCH GmbH. **c** i) Schematic illustration of the preparation and in situ self-assembly process of MSDP NPs. ii) Time-dependent NIR-II fluorescence imaging of H22 tumor-bearing mice following intravenous injection of MSDP NPs. iii) Representative tumor photographs from different treatment groups of H22 tumor-bearing mice. Reproduced with permission^191^. Copyright 2025, Wiley-VCH GmbH

Currently reported PTAs rarely exhibit PCE exceeding 70%, which, to some extent, limits the performance of PTT. Li et al. developed a NIR-II photosensitizer (FE-IDMN), which exhibited a maximum emission wavelength of 1080 nm and an ultrahigh PCE of 72.9% (Fig. 25a)^187^. FE-IDMN NPs encapsulated with DSPE-PEG2000 achieved an even higher PCE of 82.6%. Benefiting from the NIR-II emission property, FE-IDMN NPs were successfully applied in NIR-II fluorescence/PA/photothermal multimodal imaging-guided PTT. Upon 808 nm laser irradiation, the tumor site temperature was elevated to 55 °C within 5 min, thereby achieving complete ablation of 4T1 tumors.

Existing organic NIR-II materials constructed via molecular conjugation engineering or J-aggregation typically possess large molecular weights and require complex design and synthetic procedures. Lee et al. reported a short-conjugated D-A type NIR-II photosensitizer, TTP (Fig. 25b)^188^. Due to the TSCT effect between TTP monomers, Pluronic F127-encapsulated TTP NPs exhibited a significant redshift in both absorption and emission spectra, with the maximum fluorescence emission wavelength reaching 940 nm. The NPs demonstrated exceptional photophysical properties, exhibiting an 18-fold enhancement in ROS generation compared to ICG, alongside achieving a remarkable PCE of 51%. Leveraging these advantages, TTP NPs were successfully applied in NIR-II vascular bioimaging in mice and fluorescence imaging-guided tumor phototherapy. In the MCF-7 tumor-bearing mouse model, TTP NPs reached peak enrichment at the tumor site 24 h post injection. Following irradiation with an 808 nm laser for 5 min, the tumor temperature increased to 53 °C, resulting in complete tumor ablation with no recurrence observed. This study presented a facile strategy for constructing efficient NIR-II photosensitizers based on short-conjugated D-A molecular structures, thus opening up a new avenue for efficient phototherapy.

J-aggregate nanomedicines possessing in situ self-assembly capabilities hold the potential to redefine tumor targeting modalities through the assembly/aggregation-induced retention effect^240^. Li et al. developed a novel NIR-II emissive J-aggregate photosensitizer M (Fig. 25c)^191^. In aqueous solution, M tended to self-assemble into J-aggregates exhibiting remarkable NIR-II emission, with the emission wavelength extending up to 1350 nm. To enhance the stability and anti-tumor efficacy of the J-aggregates, M was co-assembled with sorafenib using DSPE-PEG2000 and DSPE-PEG2000-RGD, forming the targeted nanomedicine MSDP NPs. Under 660 nm laser irradiation, MSDP NPs demonstrated a high PCE of 45.21% and triggered pyroptosis of HepG2 cells via the generation of Type-I ROS. Furthermore, MSDP NPs successfully achieved efficient tumor penetration and sustained retention through in situ secondary self-assembly, enabling clear visualization of murine vascular structures under NIR-II imaging. Through the combination of chemotherapy and phototherapy, MSDP NPs significantly inhibited tumor growth and activated anti-tumor immune responses. This work proposed an innovative phototheranostic strategy that utilized the in situ self-assembly of NIR-II J-aggregates to induce tumor pyroptosis, offering a novel approach to overcome the limitations of existing nanomedicines in cancer therapy.

### Small-molecule metal complexes PTAs

**Fig. 26:**
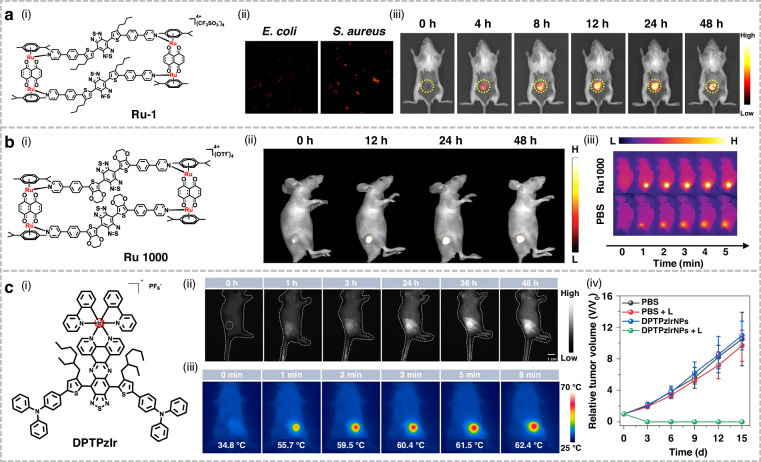
Structures and applications of metal complexes-based NIR-II molecules in phototherapy. **a** i) Structure of Ru-1. ii) NIR-II fluorescent imaging of Staphylococcus aureus and Escherichia coli at 2 h post incubation with Ru-1. iii) Time-dependent NIR-II fluorescence imaging of Staphylococcus aureus–infected wound models following intravenous injection of Ru-1 NPs. Reproduced with permission^197^. Copyright 2022, Natl. Acad. Sciences. **b** i) Structure of Ru1000. ii) Time-dependent NIR-II fluorescence imaging of A549 tumor-bearing mice following intratumoral injection of Ru1000. iii) IR thermal images of Ru1000-treated mice under different irradiation durations. Reproduced with permission^195^. Copyright 2022, Wiley-VCH GmbH. **c** i) Structure of DPTPzIr. ii) Time-dependent NIR-II fluorescence imaging of 4T1 tumor-bearing mice following intravenous injection of DPTPzIr NPs. iii) Photothermal images of DPTPzIr NPs-treated mice under different irradiation durations. iv) Tumor growth profiles from different treatment groups at day 15. Reproduced with permission^245^. Copyright 2025, American Chemical Society

The escalating challenge of antibiotic resistance necessitates novel antimicrobial strategies. PDT utilizing next-generation metal-based photosensitizers with extended excitation and emission spectra holds significant promise for antibacterial applications^16,241^. Sun et al. developed a novel Ru(II) complex (Ru-1), which exhibited a maximum emission wavelength of 1000 nm (Fig. 26a)^197^. Under 808 nm laser irradiation, it demonstrated potent ROS generation capability and significant photothermal effect. Notably, Ru-1 exhibited higher selectivity for Gram-positive bacteria, with a killing efficiency superior to that for Gram-negative bacteria. Ru-1 NPs encapsulated with DSPE-mPEG5000 prolonged the retention time in the bloodstream while maintaining robust ROS production and photothermal performance. In the mouse model infected with Staphylococcus aureus, Ru-1 NPs achieved maximum accumulation at the infection site 24 h after injection, with a fluorescence SBR as high as 7.5, successfully realizing NIR-II fluorescence imaging-guided phototherapy. This work demonstrated that long-wavelength emitting Ru (II) complexes were ideal tools for phototheranostics of pathogens, offering a promising strategy to combat drug resistance.

Despite significant progress of Ru complexes in the biomedical field, their applications in deep-tissue imaging and efficient cancer therapy are constrained by limitations such as visible-light excitation/emission properties and single chemotherapy/phototherapy modality^242,243^. Sun et al. reported a novel NIR-II emissive Ru(II) complex, Ru1000 (Fig. 26b)^195^. Benefiting from its NIR-II emission wavelength (with the maximum emission peak at 1000 nm), Ru1000 achieved a tissue penetration depth of ~6 mm. Upon 808 nm laser irradiation, the complex exhibited excellent photothermal effects and ROS generation capabilities. Notably, Ru1000 demonstrated superior cellular uptake efficiency and potent inhibitory activity against various tumor cells, including cisplatin-resistant cells. In vivo, Ru1000 enabled efficient synergistic chemo-phototherapy guided by NIR-II fluorescence imaging, demonstrating outstanding therapeutic efficacy against A549 tumors.

Owing to their high structural extensibility, tridentate Ir(III) complexes are well-suited for the design of luminescent materials with extended emission wavelengths^244^. Wang et al. reported a multifunctional Ir(III) complex, DPTPzIr (Fig. 26c)^245^. Relative to the parent ligand, DPTPzIr exhibited a significant emission red-shift to 1100 nm, accompanied by marked enhancements in ROS generation yield and PCE. DSPE-mPEG2000-encapsulated DPTPzIr NPs displayed an absorption maximum at 810 nm, demonstrating excellent spectral overlap with commercially available 808 nm laser. Moreover, DPTPzIr NPs achieved a remarkably high PCE of 60.5% and exhibited potent •OH generation capability upon laser irradiation, which was beneficial to overcoming tumor hypoxia. In 4T1 tumor-bearing murine models, DPTPzIr NPs enabled NIR-II fluorescence/PA/photothermal trimodal imaging, with the signal intensity reaching its maximum 36 h post injection. Upon 808 nm laser irradiation, the tumor temperature was effectively elevated to 59.5 °C, resulting in complete tumor ablation through the synergistic action of PDT and PTT. The development of this multifunctional metallocomplex establishes a promising avenue for advancing NIR-II phototheranostics.

## Summary and outlook

Organic small-molecule NIR-II fluorophores have emerged as a pivotal research focus in biomedical imaging and optical theranostics, owing to their facile synthesis, excellent deep-tissue penetration, high imaging contrast, and tunable spectral properties. These unique features endow them with irreplaceable potential for bridging basic research and clinical translation. This review systematically summarizes the major classes of organic small-molecule NIR-II fluorophores, including cyanine, BODIPY, BBTD, xanthene, cyano-based derivatives, and small-molecule metal complexes in the last five years. It provides in-depth discussions from the perspectives of molecular design strategies, photophysical properties, and structure-property relationships, while highlighting the latest progress in their applications in high-resolution imaging, precise surgical navigation, and multimodal imaging-guided phototherapy. Despite the rapid development of high-performance organic NIR-II fluorophores, their clinical translation remains impeded by several critical challenges:

Firstly, insufficient fluorescence intensity compromises imaging resolution. Most NIR-II fluorophores exhibit strong emission but suffer from low quantum yields. Particularly in aqueous environments, interactions between fluorophores and water molecules significantly enhance non-radiative relaxation processes, further reducing quantum yields. Currently, strategies such as incorporating bulky hydrophobic donors, forming fluorophore-protein complexes, or establishing fluorescence resonance energy transfer systems have been explored to improve quantum yields. However, achieving stable and efficient luminescence in complex physiological environments still requires further exploration.

Secondly, poor water solubility and biocompatibility restrict in vivo applications. PEGylation and nanoparticle assembly improve solubility but often lead to rapid hepatic uptake. Recent molecular-level solutions, such as zwitterionic charge balancing or compact donor-acceptor engineering, have demonstrated significantly improved pharmacokinetics. However, the trade-off between extending conjugation (for red-shifted emission) and maintaining hydrophilicity remains unresolved.

Moreover, inadequate targeting ability and specificity pose potential risks. Most NIR-II dyes operate in an “always-on” mode. Strategies based on microenvironment-activatable groups (pH, ROS, hypoxia) or conjugation with ligands (RGD, folate) have yielded improvements in SBR. Nonetheless, many systems lack quantitative comparisons of targeting efficiency, and chemical conjugation often increases synthetic complexity or alters in vivo biodistribution. Designing small-sized, intrinsically targeting or environment-specific activatable dyes remains a promising yet challenging direction.

Finally, limited BBB penetration hinders applications in central nervous system diseases. Although carrier-assisted delivery and receptor-mediated transport (e.g., GLUT1) have shown notable enhancement in BBB traversal, systematic evaluation of penetration efficiency, long-term biosafety, and clinical relevance is still scarce. Molecular designs integrating BBB-active motifs directly into the fluorophore may represent an emerging strategy with significant potential.

In summary, organic small-molecule NIR-II fluorophores have proven to be key tools in bioimaging and theranostics. Future efforts should focus on (i) rational manipulation of radiative vs. non-radiative decay pathways to boost quantum yields, (ii) compact and hydrophilic structural designs to optimize pharmacokinetics, (iii) integrating intelligent activation and precise targeting mechanisms at the molecular level, and (iv) developing BBB-permeable fluorophores supported by quantitative in vivo validation. A combination of innovative molecular engineering, systematic structure-property comparison, and rigorous biological assessment will be essential to push NIR-II fluorophores toward real clinical translation and further strengthen their role in precision medicine.
